# Brain-computer interface: an update for the clinicians

**DOI:** 10.3389/fnhum.2026.1777024

**Published:** 2026-04-22

**Authors:** Agam Jain, Sreelakshmi Raveendran, Krishnan Padmakumari Sivaraman Nair, Subasree Ramakrishnan

**Affiliations:** 1 Department of Neurology, Govind Ballabh Pant Institute of Postgraduate Medical Education and Research, New Delhi, India; 2 Department of Neurology, National Institute of Mental Health and Neurosciences, Bengaluru, India; 3 Department of Neurology, Sheffield Teaching Hospital NHS Foundation Trust Royal Hallamshire Hospital, Sheffield, United Kingdom

**Keywords:** brain computer interface, clinicians, mental health, rehabilitation, stroke

## Abstract

This narrative review critically examines the fundamental principles and clinical applications of Brain-Computer Interfaces (BCIs) in neuroscience and mental health. We searched PubMed, Scopus, and PEDro databases using pre-defined keywords, with inclusion restricted to clinical studies. The manuscript provides an evidence-based assessment of current indications, technological limitations, and emerging solutions, offering insights into both the opportunities and challenges for clinical integration. Clinical decision-making pathways are outlined to guide the adoption of BCI technologies in patient care. This article aims to increase awareness among clinicians and to equip them with the essential knowledge required as BCI systems advance toward mainstream clinical use.

## 1 Introduction

**Figure 1:**
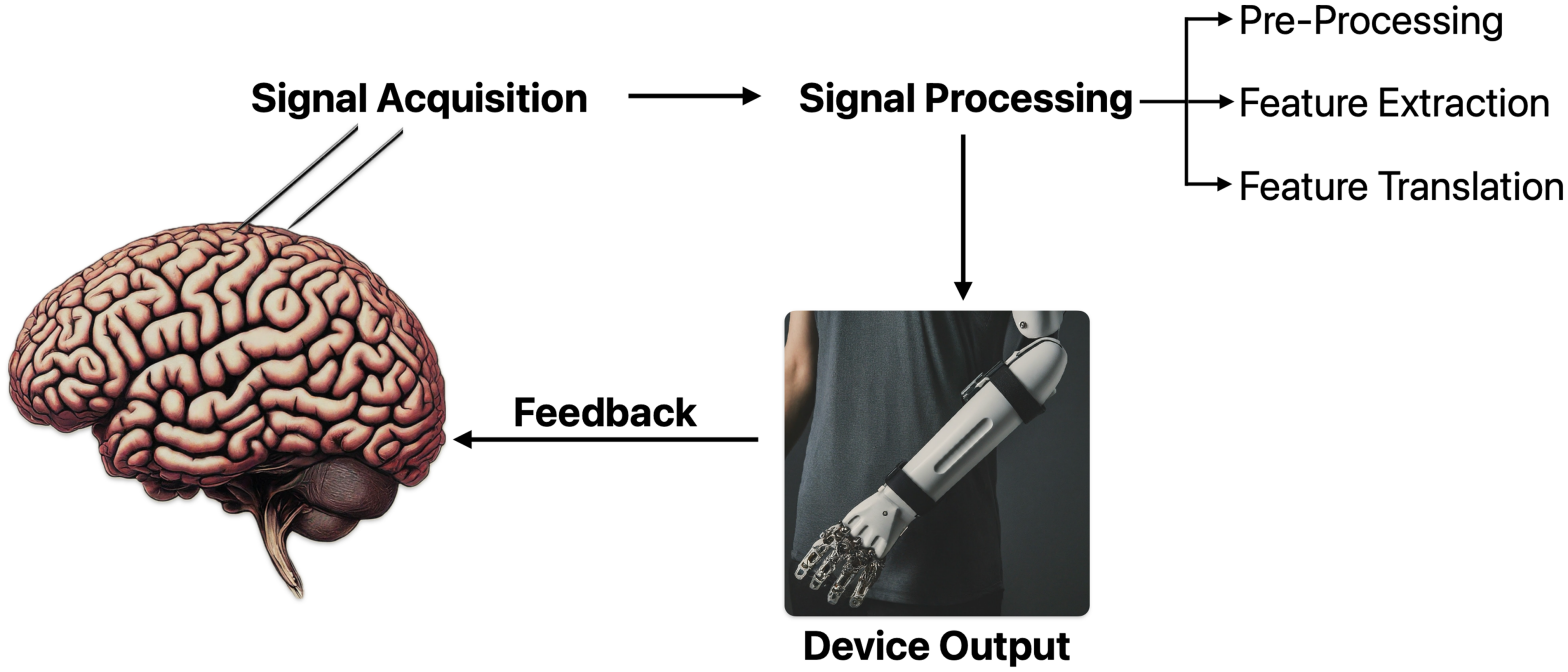
Basic outline of a brain computer interface.

Brain-Computer Interface (BCI) has been defined as “a system that measures brain activity and converts it in (nearly) real-time into functionally useful outputs to replace, restore, enhance, supplement, and/or improve the natural outputs of the brain, thereby changing the ongoing interactions between the brain and its external or internal environments. It may additionally modify brain activity using targeted delivery of stimuli to create functionally useful inputs to the brain” (BCI Definition, 2024). BCI bypasses the brain’s conventional output pathways to control external devices (Wolpaw and Wolpaw, 2012). The essential components of BCI are Signal acquisition, Signal processing (preprocessing, feature extraction and conditioning, and feature translation), and Device output (Figure 1). Feedback from the output further modulates the signal, creating a closed-loop BCI (Wolpaw and Wolpaw, 2012). While BCIs have seen rapid growth since the coining of the term BCI by Vidal in 1973, clinician knowledge about it remains low (Letourneau et al., 2020). As commercial availability rises, it is vital to demystify the field’s highly technical literature. This narrative review introduces fundamental principles of BCI to clinicians and explores various applications in clinical neuroscience. We prioritize clinical data over engineering studies and include decision-making frameworks and ethical considerations.

## 2 Methods

Computerized literature databases (PubMed, Scopus, and PEDro) were searched for peer-reviewed articles published between 1970 and January 31, 2026. Two reviewers (AJ & SR) independently conducted a literature search using predefined keywords either individually or in combination (Supplementary Box 1). We included case reports, case series, randomized controlled trials, reviews, or meta-analyses related to the clinical applications of BCI or its fundamental principles. Preclinical studies involving animals, physiology research, engineering research, and non-clinical applications were excluded. Articles retrieved from the initial search were screened by reading their titles and abstracts. Further screening involved reading the full texts of the screened studies. The resulting studies were compared between the two reviewers, and any discrepancies were resolved by a third reviewer (SreeR). Bibliographies were searched for additional relevant studies. Only articles with full-length English manuscripts were considered for the final analysis. The study screening flow is summarized in Supplementary Figure 1.

Evidence summary tables were generated for various clinical indications, except post-stroke upper limb rehabilitation and attention-deficit hyperactivity disorder, which had multiple published meta-analyses (Cervera et al., 2018; Van Doren et al., 2019; Chiu et al., 2022; Mansour et al., 2022). To create these tables, we searched the aforementioned databases for studies evaluating BCI across various clinical indications. We included all studies that reported clinical outcomes. Feasibility studies, preclinical studies, conference abstracts, and studies in foreign languages were excluded. As most studies were case series with small effect sizes and significant heterogeneity in reported outcomes, a formal risk-of-bias assessment or meta-analysis was not conducted. The Oxford Center for Evidence-Based Medicine level of evidence grading was applied (OCEBM Levels of Evidence Working Group, 2011).

## 3 Brain computer interface basics

### 3.1 Source signal

The initial step in BCI is recording brain signals. BCI can be broadly categorized into invasive, semi-invasive, and non-invasive BCI. The recorded signal might reflect a change in the electrical or magnetic field or a metabolic response. Signals vary in their ability to localize to a specific area of the brain. Spatial resolution may vary from a few centimeters (Electroencephalography) to a single neuron (intracortical electrodes). A summary of the characteristics of various source signals used for BCI is presented in Table 1 (Rao, 2013). These characteristics influence the selection of the source signal for a particular clinical indication. Additionally, various modalities may be combined to form a Hybrid BCI, for, e.g., a combination of Electroencephalography and functional near infrared spectroscopy (Pfurtscheller et al., 2010).

**Table 1:** Characteristics of various brain-computer interface source signals.

Modality	Level of invasiveness	Type of signal	Portability	Spatial resolution	Temporal resolution	Clinical research applications*	Cost
Electro-encephalography	Non-invasive	Electrical	Portable	Low	High	Motor restoration, assistive devices, spellers	Low
Magneto-encephalography	Non-invasive	Magnetic	Non-portable	Medium	High	Motor restoration	High
Functional near infrared spectroscopy	Non-invasive	Metabolic	Portable	Low	Low	Motor restoration, speller	Low
Functional magnetic resonance imaging	Non-invasive	Metabolic	Non-portable	Medium	Low	Classification in patients with disorders of consciousness, motor rehabilitation	High
Electro-corticography	Semi-invasive	Electrical	Portable	High	High	Motor assistance, speller	High
Intracortical electrodes	Invasive	Electrical	Portable	High	High	Motor & sensory assistance	High

All the above-mentioned source signals can be adapted for most clinical applications. The “Clinical Research Applications” column provides a non-exhaustive list of research applications that are commonly evaluated with the given source signal. Invasive BCIs: electrodes placed within the brain parenchyma; Semi-invasive BCIs: electrodes placed below the scalp, but not within the brain parenchyma; Non-invasive BCIs: electrodes positioned at or above the scalp.

#### 3.1.1 Electroencephalography

**Figure 2:**
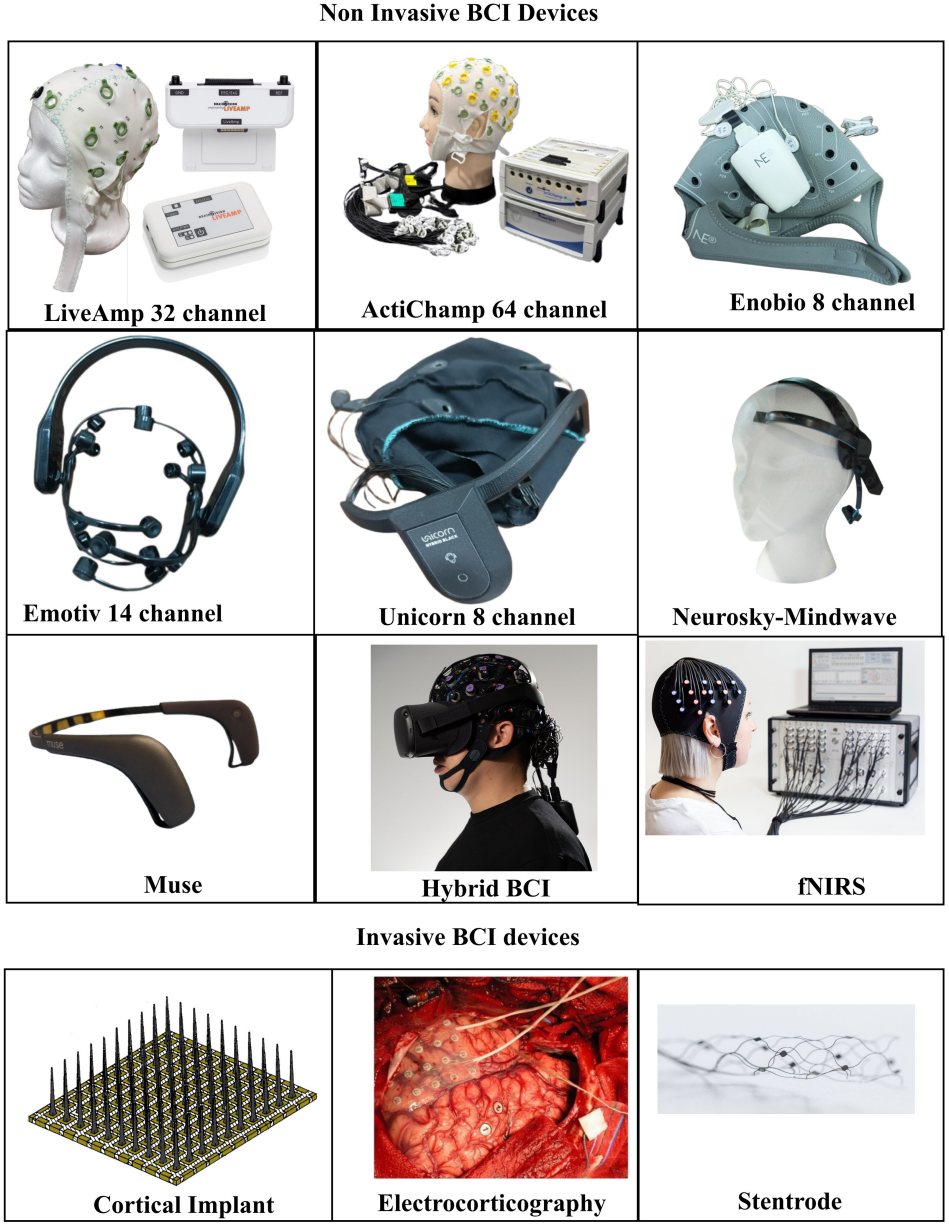
Various brain computer interface (BCI) source signal devices. LiveAmp, ActiChamp, Enobio, Emotiv, Unicorn, Neurosky, Muse are commercially available electroencephalography (EEG) devices. Hybrid BCI in this case uses electroencephalography, functional near-infrared spectroscopy (fNIRS) as the source signal, and is integrated with a virtual reality headset. Intracortical electrode image modified from: File: Utah array pat5215088.jpg. (2021, January 19). *Wikimedia Commons*. Retrieved February 15, 2026, from https://commons.wikimedia.org/w/index.php?title=File:Utah_array_pat5215088.jpg&oldid=526994679. Stentrode image from File: Stentrode Device.jpg. (2025, September 4). *Wikimedia Commons*. Retrieved February 15, 2026, from https://commons.wikimedia.org/w/index.php?title=File:Stentrode_Device.jpg&oldid=1080537674. Electrocorticography image from: File: Corticography recording.png. (2024, August 24). *Wikimedia Commons*. Retrieved February 15, 2026, from https://commons.wikimedia.org/w/index.php?title=File:Corticography_recording.png&oldid=915209505. FNIRS image from: File: Blonde fNIRS lady.jpg. (2025, March 10). *Wikimedia Commons*. Retrieved February 15, 2026, from https://commons.wikimedia.org/w/index.php?title=File:Blonde_fNIRS_lady.jpg&oldid=1008047780. Hybrid BCI image from: File: FNIRS EEG HMD.jpg. (2025, March 15). *Wikimedia Commons*. Retrieved February 15, 2026, from https://commons.wikimedia.org/w/index.php?title=File:FNIRS_EEG_HMD.jpg&oldid=1009952280.

EEG is one of the most widely used source signals because the technology is readily available, offers good temporal resolution, is cost-effective, and is portable. The drawbacks include motion or eye movement artifacts and limited spatial resolution (Rao, 2013). High-density EEG systems such as actiChamp, LiveAmp, ANTNeuro, and consumer-grade EEG systems like Emotiv, Muse, Ultracortex, Mindwave, and Unicorn Hybrid Black are frequently used in BCI studies (Figure 2) (Emotiv, 2015; Liveamp, 2017; Eego Rt, 2018; Actichamp Series, 2019; Unicorn, 2019; Jamil et al., 2021; Muse, 2024).

#### 3.1.2 Magnetoencephalography

MEG measures the tiny magnetic field perturbation caused by electrical activity in the brain. MEG offers higher spatial resolution compared to EEG. However, to measure these small signals, an elaborate setup is required. The complexity and cost of the setup limit widespread use of MEG in consumer BCI applications. Analogs of features used in EEG-BCIs have been successfully applied in MEG-BCIs (Mellinger et al., 2007; Wittevrongel et al., 2021).

#### 3.1.3 Functional magnetic resonance imaging

fMRI exploits the differences in magnetic properties of various states of hemoglobin (oxyhemoglobin is diamagnetic, whereas deoxyhemoglobin is paramagnetic). fMRI measures the blood-oxygen-level-dependent (BOLD) response, thereby estimating blood flow to a region. The fMRI response peaks 3 to 6 s after the onset of neural activity, resulting in poor temporal resolution (Sitaram et al., 2007; Glover, 2011). fMRI-based BCIs have been used in multiple scenarios such as prolonged disorders of consciousness and post-stroke upper limb rehabilitation (Owen et al., 2006; Ma et al., 2024).

#### 3.1.4 Functional near infrared spectroscopy

fNIRS measures cortical metabolic activity by emitting near-infrared light (at 690 nm and 830 nm), near the scalp. Detectors capture the back-scattered light to quantify the ratio of oxygenated to deoxygenated hemoglobin based on their differential absorption properties (Naseer and Hong, 2015). However, fNIRS is limited by poor penetration (few centimeters of the cortical surface) and low temporal resolution (hemodynamic response to neural activity takes time; Huo et al., 2023).

#### 3.1.5 Invasive and semi-invasive source signals

Invasive sensors measure electrical activity similarly to scalp EEG but offer several advantages, such as higher spatial resolution, reduced motion artifacts, and approximately 100 times higher signal-to-noise ratio (Parvizi and Kastner, 2018). Signal can be recorded from the surface of the brain (ECoG), within the brain parenchyma [intracortical electrodes, stereotactic EEG (SEEG)], or the superior sagittal sinus (Mitchell et al., 2023).

Intracortical electrodes involve the implantation of microelectrodes within the brain parenchyma. They can detect action potentials of a single neuron (single-unit activity), multiple neurons (multi-unit activity), or local field potentials. The 96-microelectrode NeuroPort array is one of the earliest and most widely used intracortical electrodes, with ample safety and efficacy data from human studies (Normann and Fernandez, 2016).

In ECoG, a strip of electrodes is implanted subdurally on the brain’s surface. In SEEG, multiple depth electrodes are implanted over the area of interest. Each electrode may have 8–18 active recording sites. ECoG and SEEG record activity from approximately 500,000 surrounding neurons and can reliably record High Frequency Broadband (HFB) range (>50 Hz) EEG (Parvizi and Kastner, 2018). HFB is believed to reflect the average spiking of surrounding neurons and to correlate with BOLD activity on fMRI during tasks (Carmichael et al., 2024).

### 3.2 Signal processing

BCIs infer user intent by processing specific signal “features” that can be reliably modulated and are stable. The pipeline begins with preprocessing to enhance signal-to-noise ratio, followed by feature extraction and conditioning (e.g., normalization) to isolate relevant data. These features are analyzed by classifiers using mathematical or machine learning models to detect patterns or threshold crossings (Lotte et al., 2007). Finally, feature translation converts these outputs into commands for devices, ranging from simple switches to complex applications like robotic arms, gait prostheses, wheelchair navigation, feedback on a computer screen or limb movement in virtual reality.

A range of features has been described for BCIs. Since EEG is one of the most used source signals, some frequently used EEG-based features are discussed below:

#### 3.2.1 Sensori-motor rhythm

**Figure 3:**
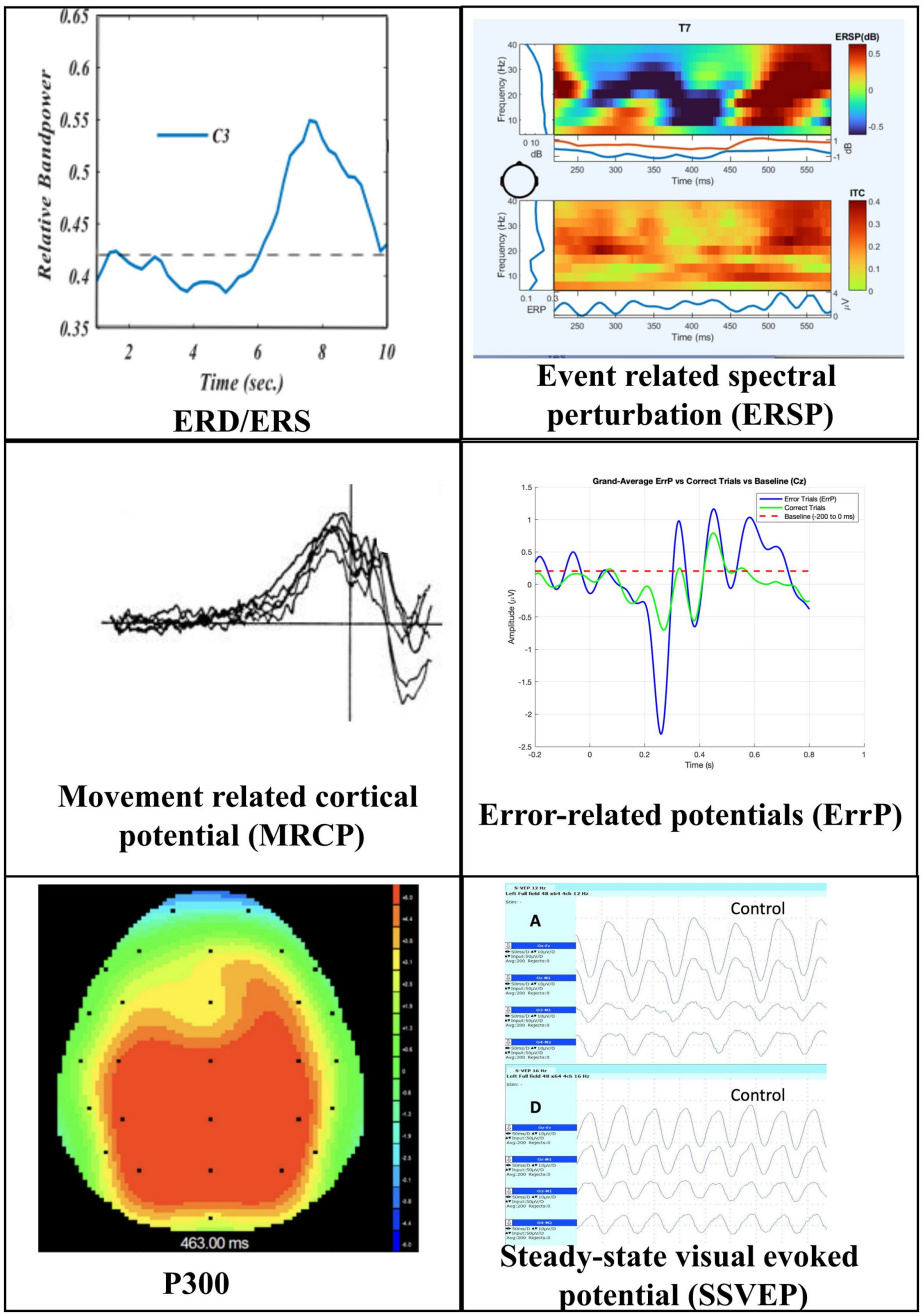
Commonly used electroencephalography features: ERD/ERS (Event-related desynchronisation/Event-related synchronization): Relative band power of the alpha band over channel C3 is plotted over time in response to a task with the right hand. Before the onset of the task, there is a decrease in bandpower (ERD), followed by an increase (ERS). ERSP (Event-related spectral perturbations): Top figure: Spectral plot, plotting the change in band power of each frequency band across time. In response to movement, the alpha band shows a decrease in power followed by an increase after movement initiation. Bottom figure: Inter-trial coherence graph plots coherence of band power across multiple trials, evaluating the consistency of the response Movement-related cortical potential: Bandpower vs. time graph over parasagittal electrode in response to a planned task summated across multiple runs. The vertical line represents movement onset. The negative drift begins a few seconds before movement onset and peaks around movement onset. Note: Image modified from: File: Bereitschaftspotenzial Fig 1.jpg. (2025, September 4). *Wikimedia Commons*. Retrieved February 15, 2026, from https://commons.wikimedia.org/w/index.php?title=File:Bereitschaftspotenzial_fig1.jpg&oldid=1080509053. Error-related potential: Band power vs. time graph. The horizontal red dotted line shows the average baseline power. Green line (change in band power in response to correct task) oscillates around baseline. Blue line (change in band power in response to incorrect task) shows a large positive deflection after task initiation. P300: Electroencephalography surface topography image, showing positive deflection over the parietal-occipital scalp (P300) around 300 milliseconds in response to an oddball event SSVEP (Steady State Visual Evoked Response): Electroencephalography traces of posterior head leads in response to a visual stimulus flashing at 12 Hertz (top image) and 18 Hertz (bottom image). Image reproduced with permission (Ahuja et al., 2020).

The electrical activity over the sensori-motor cortex is referred to as the sensori-motor rhythm (SMR). SMR can be categorized by frequency as: mu (8–12 Hz), beta (18–30 Hz), gamma (30–200 + Hz) (Wolpaw and Wolpaw, 2012). In the resting state, the mu rhythm dominates over the sensorimotor cortex. Whenever the patient makes a limb movement, the power within the mu band decreases over the contralateral sensorimotor cortex, immediately preceding the movement. This phenomenon is called event-related desynchronization (ERD). ERD begins about 2 s before the movement initiation. After the event or over the adjoining cortex, there may be an increase in power in the corresponding frequency band, called event-related synchronization (ERS; Figure 3). Wolpaw et al. described a system in 1991 that used SMR to control the vertical movement of a cursor (Wolpaw et al., 1991). SMR was also the basis for the popular Graz BCI (Scherer et al., 2007).

Interestingly, both imagining a movement (Motor imagery) and observing a movement (Action observation) elicit similar changes in SMR as performing a movement (Tani et al., 2018; Gangadharan et al., 2024). This enables SMR-based BCIs in patients with severe paralysis via motor imagery (Tani et al., 2018). Action observation has the unique advantage of being beneficial for patients with cognitive impairments who cannot actively participate in traditional physiotherapy.

#### 3.2.2 Movement related cortical potential

Movement Related Cortical Potential (MRCP) is an event-related potential observed over the centro-parietal scalp, 1.5 to 2 s before the onset of voluntary movement, reflecting motor planning (Olsen et al., 2021). Depending on what triggers the movement, it is called Bereitschafts Potential (self-triggered movement) or Contingent Negative Variation (movement in response to a cue). The MRCP waveform consists of a slow negative deflection, with the negative peak coinciding with movement initiation, followed by a positive deflection. Understandably, MRCP amplitude is greater in complex tasks. Data suggest that as one gains expertise in a given task, MRCP amplitude and latency decrease, reflecting neuroplasticity (Olsen et al., 2021).

#### 3.2.3 Evoked potentials

##### 3.2.3.1 P300

P300 refers to an event-related evoked potential characterized by a small positive deflection over the centro-parietal scalp, detectable approximately 300 ms after the stimulus (Polich, 2007). It is elicited by rare or unpredictable stimuli—the “Oddball Paradigm.” In this paradigm, a series of stimuli are presented. Whenever the patient perceives a stimulus to be rare or odd, a P300 is elicited (Figure 3). Although visual stimuli are commonly used, tactile and auditory stimuli based P300 systems have also been described (Yin et al., 2016; Guger et al., 2017).

##### 3.2.3.2 Steady state visual evoked potential

A periodic visual stimulus elicits a repeating Visual Evoked Potential (VEP) over the occipital area. The frequency of this repeating VEP matches the stimulus frequency or its harmonic (Figure 3; Vialatte et al., 2010). BCI systems utilize this by flashing multiple options at unique frequencies to identify the user’s focus. Crucially, SSVEP detection relies on mental attention rather than eye movement, remaining effective even in patients with external ophthalmoplegia (Allison et al., 2008; Lesenfants et al., 2014).

#### 3.2.4 Other features

*Slow cortical potential (SCP)* is characterized by a change in mean EEG amplitude lasting several seconds. SCP is thought to result from variations in thalamic inputs and can be modulated at will (Rao, 2013). Birbaumer *et al.* used SCP in their BCI (Thought translation device), in several patients with locked-in syndrome and amyotrophic lateral sclerosis (ALS; Birbaumer et al., 2003).

*Error-related Potential (ErrP)* is a negative potential generated over midline fronto-central electrodes, within 500 ms of the incorrect action. ErrP has a large amplitude, making it readily detectable in a single trial (Ferrez and Millan, 2008). It can be used to improve BCI accuracy (Ferrez and Millan, 2008; Pires et al., 2022).

## 4 Clinical applications of non-invasive BCI

**Figure 4:**
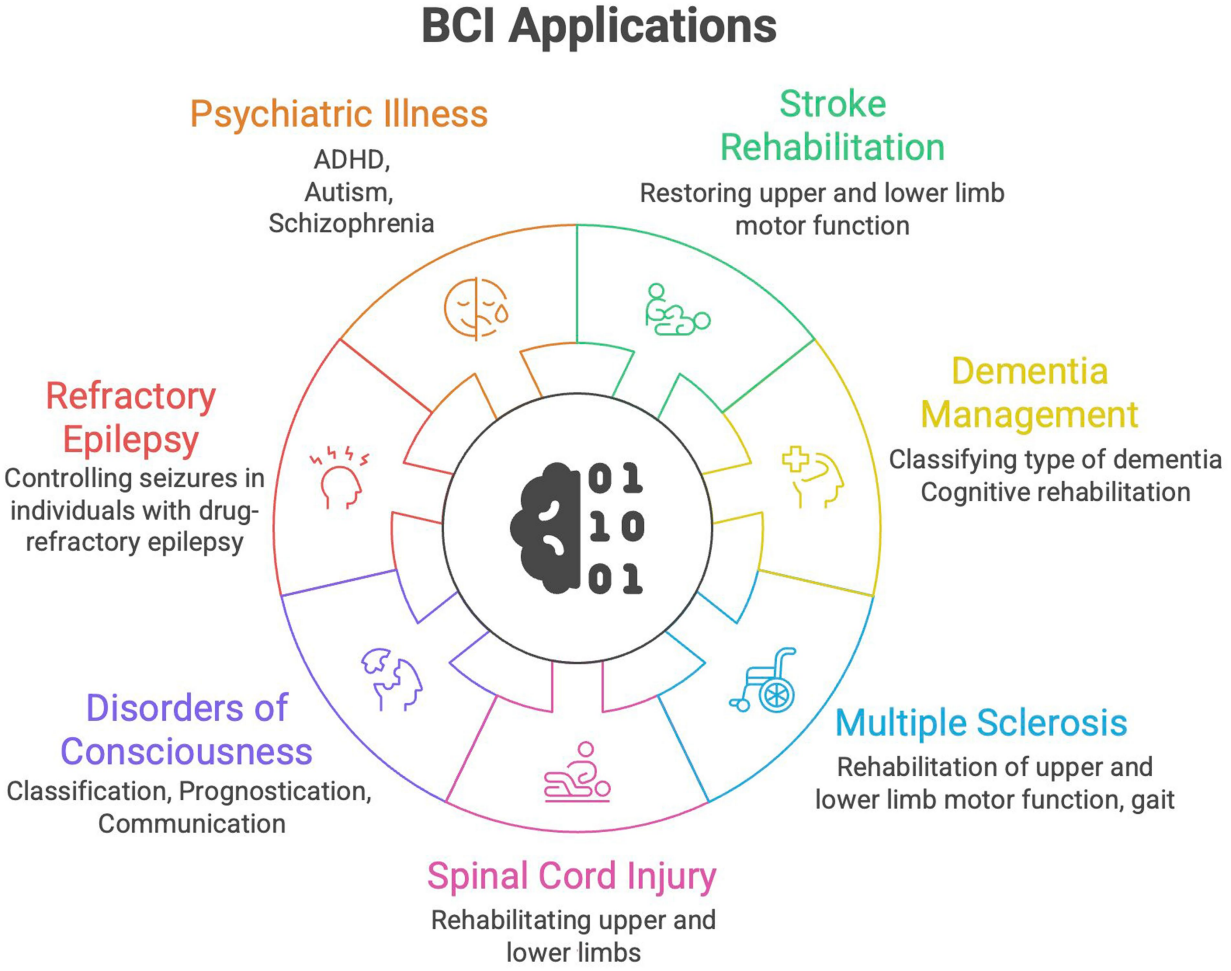
Clinical application domains of brain-computer interface (BCI, Brain Computer Interface).

BCI has been tried in patients suffering from stroke, traumatic spinal cord injury (SCI), Amyotrophic Lateral Sclerosis (ALS), Duchenne Muscular Dystrophy (DMD), Spinal-Muscular atrophy (SMA), locked-in state, disorders of consciousness, Parkinson’s disease (PD), Alzheimer’s disease (AD), Attention deficit hyperactivity disorder (ADHD), multiple sclerosis (MS), epilepsy, among others (Cincotti et al., 2008; Rao, 2013; Turconi et al., 2014; Guger et al., 2017; Utsumi et al., 2018; Benzy et al., 2020; Carrere et al., 2021; Chiu et al., 2022; Galvin-McLaughlin et al., 2022; Ortega-Robles et al., 2025). Most studies have focused on applications in motor restoration and communication. Various clinical application domains are summarized in Figure 4.

### 4.1 Motor deficits

BCIs can be used to enhance motor rehabilitation after brain injury by promoting neural plasticity (Restorative BCI) or to assist with motor activity (Assistive BCI) by controlling external devices such as wheelchairs, robotic prostheses, or orthoses.

#### 4.1.1 Restorative BCI

Motor restoration is a primary clinical application of BCI, promoting neuroplasticity through mechanisms like Neurofeedback training, Operant conditioning, Reinforcement, and Hebbian learning (Soekadar et al., 2015; Mane et al., 2020). Neurofeedback allows patients to visualize and modulate real-time brain activity, leading to lasting change in activation patterns (Sitaram et al., 2017). Operant conditioning involves rewarding the desired action, resulting in reinforcement. In the context of motor rehabilitation, motor imagery is coupled with actual limb movement facilitated by Functional Electrical Stimulation (FES) or robotic orthosis (Thompson and Wolpaw, 2015). Precise pairing of intent and movement facilitates Hebbian learning (“neurons that fire together, wire together”), strengthening the sensori-motor loop and promoting plasticity (Munakata and Pfaffly, 2004).

##### 4.1.1.1 Evidence in stroke

Most studies aiming at upper limb motor rehabilitation used an EEG BCI with SMR paradigm, coupled with limb movement via either FES, robotic orthosis, or virtual reality (VR; Ramos-Murguialday et al., 2013; Vourvopoulos et al., 2019, 2024; Khan et al., 2020; Hu et al., 2021; Kim et al., 2025). Similar studies using fNIRS as a source signal are also available (Mihara et al., 2013, 2021; Rea et al., 2014). Among the various feedback modalities, FES demonstrated the highest effect size (Mansour et al., 2022). Kruse et al. conducted a meta-analysis examining the effect of EEG-based BCI on post-stroke upper-limb rehabilitation. The data from 329 patients across 11 studies were evaluated. The BCI group demonstrated significantly higher gains in Fugl-Meyer Assessment (FMA) scores than the controls [Standardized Mean Difference (SMD) = 0.39 (0.17–0.62), *p* = 0.001] (Kruse et al., 2020). BCI improved outcomes in patients with subacute and chronic strokes, and durability up to 36 weeks has been shown (Mansour et al., 2022).

Similar studies have also been conducted to facilitate leg rehabilitation through ankle dorsiflexion, mediated either by a robotic orthosis or FES (Do et al., 2012; Xu et al., 2014; Chung et al., 2015; Zhao et al., 2022; Luo, 2024). Table 2 summarizes studies evaluating the efficacy of non-invasive BCIs for the rehabilitation of post-stroke lower-limb/gait impairment. The meta-analysis by Kruse et al. also evaluated the effect of BCI on post-stroke lower limb rehabilitation. They included two studies with a total of 32 patients, but did not find a significant difference between the two groups (Kruse et al., 2020).

**Table 2:** Summary table of studies evaluating non-invasive brain computer interface for post-stroke lower limb rehabilitation.

Author	Population (with sample size)	Intervention	Study design	Comparator	Outcome	Duration of therapy	Key findings
Randomized studies							
Chung et al. (2015)	Patients with stroke onset >6 months ago 5 in BCI, 5 in control	Sensor—EEG Feedback—foot dorsiflexion via FES	RCT	FES	Decrease in Timed Up & Go BCI = 6 s Control = 3.5 s Difference not statistically significant	5 days	Very short duration of intervention
Chung et al. (2020)	Patients with stroke onset >6 months ago. 13 in BCI, 12 in Control	Sensor—EEG Feedback– foot dorsiflexion via FES	RCT	FES	Decrease in Timed Up & Go BCI = 4.5 s Control = 4.3 s *p* = 0.946	5 weeks	Negative study in patients with chronic stroke
Yuan K. et al. (2021)	Patients with subcortical ischemic stroke within 3 months of onset with hemiplegia and cognitive impairment (MMSE<28 or MoCA<25) 16 in BCI, 14 in control	While playing VR game, Sensor—EEG Feedback– pedaling robotic orthosis	RCT	Conventional Pedaling	Increase in FMA-LE BCI = 4.5 Control = 2.1 *p* = 0.022	2 weeks	Efficacy in patients with subcortical stroke and cognitive impairment
Mihara et al. (2021)	Patients with subcortical stroke >3 months ago. 28 in BCI, 26 in control	Sensor—fNIRS Feedback—SMA neurofeedback	RCT	Sham neurofeedback	Decrease in Timed up & Go BCI = 12.84 ± 15.07 s Control = 5.51 ± 7.64 s Group difference = 7.33 s (0.83–13.83); *p* = 0.028	2 weeks	fNIRS based BCI
Zhao et al. (2022)	Patients with subcortical stroke onset within 2 weeks to 3 months. 14 patients in each group	Sensor—EEG, Feedback—movement of gait robotic orthosis and digital avatar	RCT	Movement of robotic orthosis and digital avatar not controlled by BCI	Improvement in FMA-LE BCI = 7.79 (5.90–9.67) Control = 6.29 (4.82–7.75) *p* = 0.540	4 weeks	Efficacy in patients with stroke onset within 3 months
Luo (2024)	Patients with stroke onset <2 weeks 32 in BCI, 32 in control	Sensor—EEG Feedback—foot dorsiflexion, foot inversion, calf external rotation, knee extension via FES	RCT	Conventional rehabilitation	Improvement in FMA-LE BCI = 7.74 (pre) to 13.47 (post) Control = 8.06 (pre) to 10.97 (post) *p* = <0.001	2 weeks	Efficacy in patients with stroke onset within 2 weeks. Large sample size
Wan et al. (2025a)	Patients with stroke within 1–12 months. 14 in BCI, 16 in control	While playing VR game, Sensor—EEG Feedback—pedaling robotic orthosis	RCT	Conventional pedaling	Increase in FMA-LE BCI = 4.36 ± 2.68 Control = 3.63 ± 2.85 Difference not statistically significant	4 weeks	Integration of VR, robotic pedaling. Larger RCTs required
Non-randomized studies							
(McCrimmon et al., 2014)	Patients with stroke onset >6 months ago 3 patients	Sensor—EEG Feedback—foot dorsiflexion via FES	Case Series	-	Improvement in Dorsiflexion angle by 3,4 and 8 degrees.	1 week	Improvement in physiological outcome (dorsiflexion angle) with short duration of therapy
(McCrimmon et al., 2015)	Patients with stroke > 6 months ago 9 patients	Sensor—EEG Feedback—foot dorsiflexion via FES	Case Series	-	3/9 participants had minimum detectable (10%) increase in FMA-LE score	4 weeks	Early study. Improvement in clinical parameters in chronic stroke patients
Lima et al. (2023)	Patient with hemorrhagic stroke of onset 2 months ago 1 patient	tDCS followed by BCI Sensor—EEG Feedback—foot robotic orthosis and VR	Case report	-	Increase in FMA-LE from 6 to 9	3 weeks	Integrated tDCS with BCI
(Sebastián-Romagosa et al., 2023)	Patients with stroke onset ranging from 26.7–99.66 months 22 patients	Sensor—EEG Feedback—foot dorsiflexion via FES.	Case Series	-	FMA-LE increased by 1 point, *p* = 0.166	3 months	Improvement in clinical parameters in chronic stroke patients
(Wan et al., 2025b)	Patients with stroke onset within 1–12 months 9 patients	While playing VR game, Sensor—EEG Feedback– pedaling robotic orthosis	Case series	-	Increase in FMA-LE = 4.78 ± 3.19; *p* = 0.002	4 weeks	Integration of VR, robotic pedaling.

EEG, Electroencephalography; FES, Functional Electrical Stimulation; FMA-LE, Fugl Meyer Assessment Lower Extremity; BCI, Brain Computer Interface; fNIRS, functional near infrared spectroscopy; SMA, supplementary motor area; tDCS, transcutaneous Direct Current Stimulation; VR, Virtual Reality; RCT, Randomized Controlled Trial; MMSE, Mini-Mental State Examination; MoCA, Montreal Cognitive Examination.

Multiple meta-analyses show that patients with post-stroke upper-limb impairment, who underwent combined BCI and physiotherapy, achieved better motor outcomes than those who received physiotherapy alone (Cervera et al., 2018; Bai et al., 2020; Nojima et al., 2022; Yang et al., 2022; Lin et al., 2026). BCI use was not associated with any serious adverse event (Mokienko et al., 2023). Current evidence supports BCI for post-stroke upper limb rehabilitation, though optimal patient selection criteria (e.g., demographics, stroke subtype, location, severity, and time since stroke) remain undefined. Although promising, evidence for post-stroke lower-limb rehabilitation remains insufficient. Ongoing trials may further validate the role of BCI in these patients (Biswas et al., 2024). Future research must establish optimal protocols (dosage, feedback type), investigate long-term durability, and expand targets beyond hemiparesis to other deficits seen in stroke, like dysphagia, ataxia, and cognitive impairment. Technical advancements, such as integrating cortico-muscular coherence or non-invasive brain stimulation protocols, may further improve performance (Vourvopoulos et al., 2019; De Seta et al., 2022).

##### 4.1.1.2 Evidence in Parkinson’s disease

Compared to stroke rehabilitation, clinical studies evaluating the utility of non-invasive BCIs in patients with PD are scarce. Turconi et al. coupled SMR desynchronisation to leg movement of a digital avatar using an EEG-based BCI in three patients with PD. Higher desynchronisation led to faster speed and better gait of the avatar. Over 5–6 weeks, this training improved mobility and indices of freezing of gait (Turconi et al., 2014). Subramanian et al. demonstrated improvement in PD motor scales using fMRI-based supplementary motor area neurofeedback (Subramanian et al., 2011). Studies evaluating motor rehabilitation in PD patients are summarized in Table 3. Invasive BCIs in the form of adaptive deep-brain stimulation play a central role in the management of advanced PD and are described in further sections.

**Table 3:** Summary table of studies evaluating non-invasive brain computer interface for motor rehabilitation in patients with Parkinson’s disease.

Author	Population (with sample size)	Intervention	Study design	Comparator	Outcome	Duration of therapy	Key findings
Subramanian et al. (2011)	Patients with PD H&Y—1–3 BCI—5 Control—5	Sensor—fMRI Feedback—SMA neurofeedback	Case Series	Motor imagery	Improvement in mean UPDRS motor function: BCI = 14.2 (pre) to 9 (post); *p* = 0.042 Control = 15 (pre) to 13.4 (post); *p* = 0.336	2–6 months	fMRI based BCI, significant improvement in UPDRS
Buyukturkoglu et al. (2013)	1 Patient with PD H&Y—2.5	Sensor—fMRI Feedback—SMA neurofeedback	Case report	-	Increase in mean motor response time: Pre = 4,260 ± 987 msec Post = 4,283 ± 1,070 msec	2 days	Very short duration of intervention
Turconi et al. (2014)	3 patients with PD & Freezing of gait H&Y—1.5–2.5	Sensor—EEG Feedback—walking speed of digital avatar linked to SMR desynchronization	Case Series	-	Improvement in FOGQ (2 to 3 points), MPAS (2 to 4 points), Berg Balance scale (−2 to 3 points), Timed up and Go (0.92 s to 1.97 s)	5–8 weeks	Targeted freezing of gait, balance

EEG, Electroencephalography; PD, Parkinson’s Disease; H&Y, Hoehn and Yahr scale; SMR, Sensori-motor rhythm; fMRI, functional magnetic resonance imaging; BCI, Brain Computer Interface; SMA, supplementary motor area; RCT, Randomized Controlled Trial; FOGQ, freezing of gait questionnaire; MPAS, modified Parkinson activity scale; UPDRS, unified Parkinson disease rating scale.

Despite multiple studies exploring the feasibility of various non-invasive BCI paradigms in PD, data regarding clinical efficacy remain limited. Current evidence does not support the clinical use of non-invasive BCIs in patients with PD. Unlike static insults such as stroke and traumatic brain injury, neurodegenerative diseases like PD may need multiple recalibrations and therapy sessions. Future research should target various motor symptoms (bradykinesia, rigidity, tremor, motor fluctuations, postural instability, gait, freezing), Hoehn and Yahr stages, and non-motor manifestations while also extending to other types of Parkinsonism (Progressive supranuclear palsy, multi-system atrophy, vascular parkinsonism).

##### 4.1.1.3 Evidence in spinal cord injury

EEG-based BCIs with feedback via FES, robotic orthosis, or VR have shown promise for improving motor and non-motor symptoms in patients with SCI (Osuagwu et al., 2016; Nicolelis et al., 2022; Pais-Vieira et al., 2024; Li et al., 2025). Table 4 summarizes clinical studies aimed at motor rehabilitation of patients with SCI using non-invasive BCI. In a meta-analysis of 9 studies and 109 SCI patients, non-invasive BCI led to improvement in motor (SMD = 0.72; *p* < 0.01), sensory (SMD = 0.95; *p* < 0.01), and activities of daily living (SMD = 0.85; *p* < 0.01; Sun et al., 2025). Invasive BCIs have been used to bypass damaged sections of the cord for assistive and restorative purposes. Invasive BCIs are discussed in greater detail in a later section.

**Table 4:** Summary table of studies evaluating non-invasive brain computer interface for motor rehabilitation in patients with spinal cord injury.

Author	Population (with sample size)	Intervention	Study design	Comparator	Outcome	Number of therapy sessions	Key findings
Upper limb rehabilitation							
Osuagwu et al. (2016)	Traumatic SCI C4-C7 AIS B-C <3 months since injury BCI—7 patients Control—5 patients	Sensor—EEG Feedback –hand movements via FES	RCT	FES triggered hand movement independent of EEG	Significant improvement in MMT score in BCI group but not in control	20 sessions over 4–5 months	Demonstrated clinical efficacy in patients with injury within 3 months
Jovanovic et al. (2021)	Traumatic SCI C4-C7 AIS B-D <6 months since injury BCI—5 patients	Sensor—EEG Feedback—functional hand movements via FES	Case Series	-	FIM self care: Mean increase 10.0 ± 8.16 (for 3 patients who completed end of therapy assessment) SCIM self-care: Mean increase 7.33 ± 5.73	12–40 sessions over 12 weeks	Functional independence scores rather than motor scores as main outcome
Cantillo-Negrete et al. (2023)	Traumatic SCI C4-C7 AIS A-D >6 months since injury BCI-6 patients	Sensor—EEG Feedback –hand movements via FES	Case Series	-	2 patients achieved MCID in ARAT in left hand 3 patients achieved MCID in ARAT in right hand 2 patients achieved MCID in UEMS	12 sessions over 4 weeks	Improvement in hand motor scores in patients with chronic SCI
Lower limb rehabilitation							
**Randomized studies**							
Nicolelis et al. (2022)	Traumatic SCI T4-T7 AIS A-B 6 months—3 years since injury BCI—4 patients Control—4 patients	Sensor—EEG Feedback –movement of 3D avatar plus tactile	RCT	Locomotor Training	Increase in LEMS BCI = 3.5 Control = 2.5 *p*-value not provided	14 weeks	Tested tactile + digital feedback. Trend toward improvement in motor scores
Hu et al. (2026)	Traumatic SCI C4-C7 AIS B-C <3 months since injury BCI—10 patients Control—11 patients	Sensor—EEG Feedback –Robotic orthosis	RCT	Robotic Orthosis	Increase in LEMS = BCI: 20.8 ± 4.1 to 22.2 ± 4.0; *p* = 0.003 Control: 21.1 ± 4.3 to 21.6 ± 4.2; *p* = 0.059	4 weeks	Significant improvement in clinical outcome with robotic orthosis
**Non-randomized studies**							
Donati et al. (2016)	Traumatic SCI T4-T11 AIS A-B 3–13 years since injury BCI-8 patients	Sensor—EEG Feedback –Robotic gait orthosis + VR	Case series	-	AIS improved from A to C in 3 AIS improved from B to C in 1	12 months	Landmark study demonstrating efficacy in chronic SCI with long term training
Shokur et al. (2018)	Traumatic SCI T4-T11 AIS A-B 3–13 years since injury BCI-8 patients Same set of patients as Donati et al. (2016)	Sensor—EEG Feedback –Robotic gait orthosis + VR	Case series	-	AIS improved from A to C in 6 AIS improved from B to C in 1 LEMS data not available at baseline	28 months	Landmark study demonstrating efficacy in chronic SCI with long term training
Selfslagh et al. (2019)	Traumatic SCI T4-T7 AIS C 4.5–10 years since injury BCI-2 patient	Sensor—EEG Feedback –FES based gait training	Case series	-	Increase in LEMS – Patient 1 = 3 Patient 2 = 9	22 sessions over 5 months	FES based training over months improved motor scores
Cui et al. (2022)	Traumatic SCI C7-L2 AIS A-C 4–30 months since injury BCI-7 patients	Sensor—EEG Feedback –Robotic orthosis	Case series	-	Mean increase in LEMS = 2.6 ± 0.8 AIS improved from A to C in 1 AIS improved from B to C in 3	4–7 weeks	Robotic orthosis based training over weeks improved motor scores

EEG, Electroencephalography; FES, Functional Electrical Stimulation; FMA-LE, Fugl Meyer Assessment Lower Extremity; BCI, Brain Computer Interface; VR, Virtual Reality; RCT, Randomized Controlled Trial; SCI, Spinal Cord Injury; AIS, American Spinal Injury Association Impairment Scale; FIM, Functional Independence Measure; SCIM, Spinal Cord Independence Measure; MMT, Oxford Manual Muscle Test; ARAT, Action Research Arm Test; UEMS, Upper Extremity Motor Score; MCID, Minimal clinically important difference; LEMS, Lower Extremity Motor Score.

Current studies are limited by a small sample size and the lack of blinding among patients or healthcare providers. Furthermore, the studies differ significantly in terms of time since injury and injury severity. Although promising, current evidence does not support the routine clinical use of non-invasive BCIs for patients with SCI. Additionally, transcutaneous spinal cord stimulation alone has demonstrated encouraging results (Moritz et al., 2024). Combining transcutaneous spinal cord stimulation with BCI may improve efficacy and warrant further investigation. Future studies should broaden their focus to include non-traumatic myelopathies, generate high-quality evidence, and categorize patients based on the etiology, severity, and time since the injury.

##### 4.1.1.4 Evidence in multiple sclerosis

Despite the advent of multiple highly effective drugs, MS often results in multi-domain disability. Although many studies have demonstrated feasibility in patients with MS, very few have evaluated clinical benefits. Carrere et al. investigated BCI-FES paradigm in seven patients with MS without enrolling any controls. After 8 weeks of therapy, there was a significant improvement in gait speed (Carrere et al., 2021). Clinical studies for motor rehabilitation in MS patients are summarized in Table 5.

**Table 5:** Summary table of studies evaluating non-invasive brain computer interface for gait rehabilitation in patients with multiple sclerosis.

Author	Population (with sample size)	Intervention	Study design	Comparator	Outcome	Duration of therapy	Key findings
Carrere et al. (2021)	9 patients with MS with EDSS 4.5–6.5	Sensor—EEG Feedback—ankle dorsiflexion FES	Case Series	-	Decrease in median T25FW time from 9.62 s to 7.63 s (*p* = 0.018)	8 weeks	In MS patients with gait impairment, BCI therapy improved walking speed
Sebastián-Romagosa et al. (2025)	24 patients with MS with EDSS≤6.5	Sensor—EEG Feedback—FES, VR avatar	Case Series	-	Increase in 6MWT distance = 37.3 m (21.5–53.1); *p* < 0.001 Log ratio After/Baseline of T25FW time = 0.873 (0.77–0.9); *p* < 0.001	2.5 months	Improvement in walking speed with combination of FES and VR

EEG, Electroencephalography; FES, Functional Electrical Stimulation; MS, Multiple Sclerosis; EDSS, Expanded Disability Status Scale; VR, Virtual Reality; 6MWT, 6 min walking test; T25W, Timed 25 Foot Walk.

Current evidence does not support the routine clinical use of BCI in MS patients. MS presents with unique challenges, such as the development of new deficits, disruption of white matter integrity over time, and disabling fatigue. Additional research is needed to explore the role of BCI in MS subtypes, and in other demyelinating diseases, such as neuromyelitis optica, myelin oligodendrocyte glycoprotein antibody-associated disease, and secondary demyelinating illnesses.

##### 4.1.1.5 Evidence in amyotrophic lateral sclerosis

Multiple studies have explored the feasibility of BCIs for this progressive motor disorder (Kübler et al., 2005; McCane et al., 2015; Hosni et al., 2020). Non-invasive BCI use in this population has been restricted to demonstrations of assistance in activities of daily living through robots, orthoses, cursor control, environment control, and speech assistance via spellers in patients with locked-in state (Bai et al., 2010; Schettini et al., 2015; Spataro et al., 2017). Further details about these applications are discussed in the section on Assistive BCI. There is a lack of studies on non-invasive BCIs aimed at restoring motor function in ALS. Current evidence does not support the use of BCIs for motor restoration in patients with ALS.

##### 4.1.1.6 Commercially available non-invasive BCI for motor rehabilitation

An EEG-based rehabilitative BCI called RecoveriX has demonstrated remarkable results in the rehabilitation of upper and lower limbs in patients with stroke and MS. It employs a motor imagery paradigm and provides feedback using Functional Electrical Stimulation (FES) or VR (Irimia et al., 2016; Sebastián-Romagosa et al., 2020, 2023). The device is being investigated for its role in PD, Guillain-Barré Syndrome (recoveriX, 2024a,b). Another comparable product is Ipsihand. In 2021, it received approval from the United States Food and Drug Administration (FDA) for post-stroke upper limb rehabilitation. It uses SMR paradigm coupled to hand movement using a robotic orthosis. Interestingly, Ipsihand uses signals generated from the contralesional cortex (Rustamov et al., 2023).

#### 4.1.2 Assistive BCI

BCI can be used to operate mobility devices (wheelchairs, prosthetic limbs, exoskeletons), communication devices, or environmental control systems, smart household appliances (Aloise et al., 2011; Gancet et al., 2012; Contreras-Vidal and Grossman, 2013; Do et al., 2013; Kosmyna et al., 2016; Meng et al., 2016; Waytowich and Krusienski, 2017; Rashid et al., 2021). A notable study from Spain demonstrated the feasibility of using MRCP-based BCI in controlling an exoskeleton to support ambulation in patients with paraparesis secondary to traumatic SCI (López-Larraz et al., 2016). Web browsers explicitly designed for BCI use have also been described (Yin et al., 2009).

Mobility and environmental control BCIs show promise and may enhance patients’ quality of life and independence, and reduce caregiver burden. Despite multiple proof-of-concept studies, studies demonstrating real-world usefulness in patients over a meaningful duration are scarce. Currently, such devices are restricted to laboratory settings and are not yet suitable for routine clinical use (Colucci et al., 2022). Rigorous safety assessments against signal misclassification are essential before clinical adoption. Exoskeleton designs should also account for anatomical variation among patients to ensure proper alignment of actuators at the joint. Spasticity, contractures, and restricted range of motion at various joints increase the complexity of operating these exoskeleton devices.

### 4.2 Communication

BCI can aid communication among patients with locked-in state or anarthria (Sellers et al., 2014). Various non-invasive speller designs have been tested among patients with ALS, MS, DMD, and SMA (Naito et al., 2007; Cincotti et al., 2008; Utsumi et al., 2018; Wolpaw et al., 2018; Riccio et al., 2022). Wolpaw et al. deployed P300-based spellers at home in patients with anarthria secondary to ALS for over a year. The patient and the caregiver reported that the system’s benefits outweighed the burden (Wolpaw et al., 2018).

Farwell and Donchin described a P300-based speller in 1988. Users were presented with a 6 × 6 matrix of letters and commands, with rows and columns highlighted in random order (Farwell and Donchin, 1988). Whenever the row/column containing the desired letter was highlighted, a P300 would be elicited (Oddball paradigm), selecting the row/column. Another speller design is Hex-o-spell, which is an SMR-based speller (Blankertz et al., 2006). The patient is presented with six hexagons arranged in a circle around a central needle. The imagination of right-hand movement is linked to needle rotation. The imagination of foot movement stops the needle and selection of the adjoining hexagon. In a typical SSVEP-based speller, the user is presented with four boxes at the corners of the screen. Each box, containing a matrix of letters/numbers, flashes at a unique frequency. The desired square is selected by comparing its frequency to SSVEP (Li et al., 2021).

Design variations can improve performance (superimposing alphabets on the face of a famous/familiar face; coloring the face green; coloring rows in separate colors—“Chroma Speller”; displaying characters in checkerboard or text on nine keys format; varying the size of the target box with detection certainty; Rezeika et al., 2018; Fodor et al., 2024). Code-modulated Visual Evoked Potential (cVEP) offers improved accuracy, speed, and numerous possible selections without the need for calibration (Martínez-Cagigal et al., 2021). Steady-State motion Visual Evoked Potentials (SSmVEP) and cVEP have lowered BCI illiteracy rates (Volosyak et al., 2020). A few devices, such as the Unicorn Hybrid Black Assistive Speller, are commercially available (Unicorn, 2019). Similar fNIRS-based devices have also been described (Naito et al., 2007). Invasive BCIs for similar purpose are described in detail in a later section (Pandarinath et al., 2017).

Even with the highest reported Information Transfer Rate (ITR) of 360 bits/min, BCI spellers remain slow for everyday use (Yao et al., 2018). Marchetti et al. conducted a systematic review and meta-analysis of the effectiveness of P300 speller devices in patients with ALS. The pooled classification accuracy was 74%, which may not be sufficient for routine clinical use. The review was limited by a lack of RCTs and substantial heterogeneity among reported case series (Marchetti and Priftis, 2014). Incorporating word prediction, machine learning, and hybrid BCI designs may improve performance and ITR. Most speller designs have been tested in healthy individuals and require more rigorous evaluation across different disease subtypes. The existing evidence is limited to case series. Additionally, BCI-based spellers should be compared to state-of-the-art assistive communication devices to assess their true clinical utility. The current evidence does not support the routine clinical use of BCI-based spellers. They may be considered for patients who are unable to use traditional assistive communication devices.

#### 4.2.1 Aphasia rehabilitation

Few studies have explored the role of BCI in the rehabilitation of people with aphasia (Kleih et al., 2016; Musso et al., 2022; Kleih and Botrel, 2024). Musso et al. designed an auditory P300 paradigm, in which the patient is presented with a sentence, with the last word missing. Subsequently, a list of words is presented, with the target word among the options. Whenever the patient hears the target word, a P300 is generated, selecting the word. At the end of each selection, the patient receives feedback regarding the correct choice. This paradigm was tested in 10 stroke patients. Post-training Aachen Aphasia Test scores showed significant improvement (Hedges g_s_ = 0.52; *p* = 0.007; Musso et al., 2022). Currently, evidence supporting the role of BCI in aphasia rehabilitation is limited to a few case series and does not support routine clinical use. Further studies are needed to explore the utility of BCI in aphasia rehabilitation.

### 4.3 Prolonged disorders of consciousness

Patients with PDOC are classified as unresponsive wakefulness (UWS) or minimally conscious state (MCS) based on bedside evaluation using scales such as the Coma Recovery Scale-revised (CRS-R). Some patients can perform tasks outlined on CRS-R using a BCI, despite not being able to perform the same task as a behavioral response. Such patients may have preserved abilities to follow commands, perform mental imagery, understand syntactically complex sentences, differentiate words from pseudo-words, or recognize factually correct or incorrect sentences (Aubinet et al., 2022; Galiotta et al., 2022). This covert consciousness is often underrecognized, leading to underestimation of prognosis and therapeutic nihilism.

BCI can be useful for classification, prognostication, communication, and rehabilitation of PDOC patients (Lulé et al., 2013; Pan et al., 2014; Guger et al., 2018; Spataro et al., 2018; Annen et al., 2020; Murovec et al., 2020). Patients are often stimulated with auditory, visual, vibro-tactile, or multi-modal stimuli, and changes in brain activity are monitored. Studies have demonstrated that up to 33% of UWS patients can generate cortical responses to language stimuli, and 20% can follow commands. The percentages are much higher (50 and 33%, respectively) among MCS patients without clinically observable language responses (Aubinet et al., 2022). Patients who can use BCIs above chance level may have a better prognosis (Pan et al., 2014; Spataro et al., 2018).

The BCI paradigms must be designed to accommodate fluctuating alertness, limited cognitive abilities, and variable sensory deficits across patients. Current studies are limited by small sample sizes, heterogeneous stimulation protocols, time since injury, and assessment methods. Large-scale clinical studies, along with standardized BCI-based protocols for assessment and classification of PDOC patients, are urgently needed. Mindbeagle is a commercially available BCI for assessment, communication, and prognostication in patients with PDOC (Mindbeagle, 2015). Current evidence supports the role of BCI as an adjunct in classifying patients with PDOC.

### 4.4 Dementia

Changes in neural correlates (measured by EEG or fNIRS) in response to a cognitive task have been used to differentiate Mild Cognitive Impairment (MCI) from dementia or classification of dementia subtypes (Cicalese et al., 2020; Trinh et al., 2021; Perez-Valero et al., 2022; Rutkowski et al., 2024). BCI has been used as a neurofeedback tool for cognitive rehabilitation among patients with dementia, stroke, and traumatic brain injury (Ali et al., 2020; Kaimara et al., 2020; Sun et al., 2022; Liu et al., 2023; Tazaki, 2024; Suhail et al., 2025). In a randomized trial by Jirayucharoensak et al. patients with MCI who received EEG-based neurofeedback had significant improvement in rapid visual processing and spatial visual memory (Jirayucharoensak et al., 2019). Clinical studies aiming at cognitive rehabilitation using non-invasive BCIs across various diseases are summarized in Table 6. VR-based immersive environment, olfactory, affective, and facial emotion recognition paradigms are also being explored in this population (Wen et al., 2021; Morozova et al., 2023; Wu et al., 2023).

**Table 6:** Summary table of studies evaluating non-invasive brain computer interface for cognitive rehabilitation in patients with various diseases.

Author	Population (with sample size)	Intervention	Study design	Comparator	Outcome	Duration of therapy	Key findings
Randomized studies							
Kober et al. (2015)	Patients with stroke onset >1 month BCI: 17 patients Controls: 7 patients	Sensor—EEG SMR or Upper alpha neurofeedback training	RCT	Conventional care	SMR group—significant improvement in verbal short term and long term memory scores Upper alpha group—significant improvement in verbal short term memory scores Controls—no change	10 sessions	Neurofeedback training led to improvement in language and memory domains in patients with VCI
Jirayucharoensak et al. (2019)	Neurofeedback: 32 MCI patients and 26 healthy elderly women Exergame: 19 MCI patients and 17 healthy elderly women Usual care: 14 MCI patients and 11 healthy elderly women	Sensor—EEG Neurofeedback	RCT	Exergame (Cognitive training game) Usual Care	Neurofeedback improved rapid visual processing, spatial working memory	20 sessions over 3 months	BCI superior to cognitive game. Testing in MCI patients
Yuan Z. et al. (2021)	Patients with subcortical stroke 1 week to 3 months ago MMSE<28 MoCA<25 BCI—16 patients Control—14 patients	Sensor—EEG Feedback—robotic pedaling orthosis and VR	RCT	Conventional pedaling based exercises	Improvement in MMSE and MoCA in both groups, no statistically significant difference between groups	2 weeks	Tested short duration of therapy in patients with stroke onset within 3 months. BCI based motor training improved cognitive outcomes.
Zhao et al. (2022)	Patients with stroke within 2 weeks to 3 months BCI—14 patients Control—14 patients	Sensor EEG Feedback Robotic orthosis mediated movement	RCT	Conventional physiotherapy	Improvement in cognitive score (LOCTA) BCI: 26.86 vs. Control: 14.86; *p* = 0.049	4 weeks	BCI based motor training improved cognitive outcomes.
Annaheim et al. (2022)	Patients with severe frontal or fronto-temporal brain lesions BCI—10 patients Control—10 patients	Sensor—EEG Infralow frequency neurofeedback	RCT	Sham	No significant difference between two groups	16 sessions	Patients with non traumatic acquired frontal lobe lesions also included. Unequal distribution of traumatic and non traumatic etiology in BCI and control group
Liu et al. (2023)	Patients with stroke within 1 month with attention component of MoCA 2–5 BCI—30 patients Control—30 patients	Sensor—EEG Motor imagery contingent limb movement mediated by FES	RCT	Conventional rehabilitation	Significant Improvement in BCI vs. controls in EEG correlates of attention: Alert network response time (Group difference = 32.4 ms; 95% CI, 58.4 to 85.6; *p* < 0.001); Orienting network response time (Group difference = 5.6 ms; 95% CI, 29.8 to 55.8; *p* = 0.010)	3 weeks	Included patients with stroke onset within 1 month. Short duration of therapy
Non-randomized studies							
Luijmes et al. (2016)	10 patients with AD	Sensor—EEG Neurofeedback	Case Series	-	No improvement or decline in cognitive score	15 weeks	Testing in AD patients
Kübler et al. (2017)	Patients with stroke Subacute stroke—12 patients Chronic stroke—15 patients	Sensor—EEG SMR & SCP based Neurofeedback	Case series	-	Patients who learned to control SMR showed improvement in cognitive scores	8–10 sessions	VCI patients who could modulate SMR/SCP had improvement in cognitive scores
Kober et al. (2017)	1 patient with SAH and parenchymal hemorrhage 1 patient with stroke 24 healthy elderly	Sensor—EEG Upper Alpha Neurofeedback training	Case Series	-	Patient with SAH—significant improvement in short term and long term memory score as compared to baseline Patient with MCA infarct—significant improvement in cognitive flexibility and long term memory scores as compared to baseline. Healthy elderly—no significant change	10 sessions over 2–3 weeks	Efficacy of short duration neurofeedback therapy in patient with SAH
Kober et al. (2019)	14 Patients with RRMS or SPMS	Sensor-EEG SMR based Neurofeedback	Case Series	-	7 patients showed improvement in cognitive domains (long term memory, executive function) as measured by Brief Repeatable Battery of Neuropsychological Tests (BRB-N) Patients with cognitive improvement could modulate SMR during neurofeedback	4 weeks	Testing in MS patients. Ability to modulate SMR associated with improvement in cognitive outcomes
Kotov et al. (2020)	Patients with stroke onset 2 months to 2 year BCI—22 patients Control—22 patients	Sensor—EEG Feedback—Robotic hand orthosis	Non-randomized trial	Conventional rehabilitation	Improvement in MoCA BCI = 24.81 ± 0.35 to 26.68 ± 0.40; *p* = 0.0002 Control = 24.45 ± 0.68 to 25.64 ± 0.67; *p* = 0.0015	8–10 sessions	BCI based motor training improved cognitive outcomes.
Pinter et al. (2021)	14 Patients with RRMS or SPMS Same group of patients as (Kober et al., 2019)	Sensor-EEG SMR based Neurofeedback	Case Series	-	7 patients showed improvement in cognitive domains (long term memory, executive function) as measured by Brief Repeatable Battery of Neuropsychological Tests (BRB-N) Patients with cognitive improvement could modulate SMR during neurofeedback Cognitive improvement correlate with Functional connectivity in Salience Network	4 weeks	Testing in MS patients. Cognitive improvement associated with changes in connectivity and micro-structure
Cripe et al. (2021)	Retrospective chart review 200 patients with closed head injury	Restorative cognitive remediation training Sensor—EEG BCI mediated difficulty adjustment based on cognitive load and task engagement	Case Series	-	Improvement in cognitive assessment scores (Woodcock Johnson III)	12 weeks	Testing in traumatic brain injury patients. Large sample size
Galvin-McLaughlin et al. (2022)	2 patients with mild AD (CDR 0.5–1; MoCA> = 14; MMSE> = 18)	Sensor—EEG Neurofeedback	Case Series	-	Mixed results	6 weeks	Testing in AD patients
Kleih-Dahms and Botrel (2023)	5 patients with stroke > 6 months	Sensor—EEG Slow Cortical Potential Neurofeedback	Case Series	-	Mixed results	20 sessions	Used slow cortical potentisl neurofeedback
Lee et al. (2023)	13 patients with MCI	Sensor—fNIRS Neurofeedback	Case Series	-	Significant improvement in working memory scores: Boston naming test (13.08 vs. 12.15; *p* = 0.023), word list recognition (9.23 vs. 8.62; *p* = 0.027), construction recall (8.08 vs. 6.62; *p* = 0.02), forward digit span (8.76 vs. 7.23; *p* = 0.032)	4 sessions over 4 weeks	Testing in MCI patients. fNIRS based BCI
Chen et al. (2025)	Patients with stroke onset >1 month BCI—31 patients Only FES—35 patients Control—34 patients Long term (4.5 year) follow-up BCI—12 patients Only FES—12 patients	Sensor—EEG Feedback—FES mediated limb movement	Case Series	FES only Conventional Physiotherapy	Mean improvement in MoCA at 4 months: 1.6 (BCI) vs. −0.2 (FES) vs. 0.6 (Control)	8 weeks	BCI based motor training improved cognitive outcomes.
Suhail et al. (2025)	Stroke—6 patients MCI—7 patients Healthy controls—2	Sensor—EEG Feedback—Attention based neurofeedback game	Case series	-	Improvement in EEG based attention scores Stroke—by 4.29–32.18% MCI—by 4.32 to 48.25% 1 Healthy control showed improvement by 12.99%	3 days	Testing in MCI and VCI patients. Very short duration of therapy
Wan et al. (2025b)	12 patients with stroke onset 1–12 months ago	Sensor—EEG Feedback—robotic pedaling orthosis and VR	Case series	-	Improvement in MoCA: 1.89 ± 2.37; *p* = 0.044	4 weeks	BCI based motor training improved cognitive outcomes
Cao et al. (2025)	5 patients with stroke onset within 6 months	Sensor—EEG Feedback—Robotic orthosis	Case Series	-	Average MMSE increased from 17.5 to 21.8	6 weeks	BCI based motor training improved cognitive outcomes

EEG, Electroencephalography; FES, Functional Electrical Stimulation; BCI, Brain Computer Interface; fNIRS, functional near infra-red spectroscopy; VR, Virtual Reality; RCT, Randomized Controlled Trial; MMSE, Mini mental state examination; MoCA, Montreal Cognitive Examination; RRMS, Relapsing Remitting Multiple Sclerosis; SPMS, Secondary Progressive Multiple Sclerosis; SMR, Sensori-motor rhythm; SCP, Slow Cortical Potential; SAH, subarachnoid hemorrhage; MCA, Middle cerebral artery; MCI, mild cognitive impairment; CDR, Clinical dementia rating scale; LOCTA, Loewenstein Occupational Therapy Cognitive Assessment; VCI, Vascular Cognitive Impairment; MS, Multiple Sclerosis.

There is considerable heterogeneity in the literature regarding the cognitive disorders tested, the interventions employed, and the assessment scales used. The reported studies have small sample sizes, lack controls, require long-term follow-up, and are subject to reporting bias (Ali et al., 2020). Improvements in cognitive testing secondary to learning and doubts about the translation of cognitive score gains into real-world benefits are additional concerns in these disorders (Peckham, 2021). BCI, for cognitive rehabilitation in the form of neurofeedback, has been studied extensively in healthy elderly. Research involving patients remains limited. The evidence is not sufficient to recommend routine clinical use of BCI for cognitive rehabilitation or for classifying dementia subtypes. BCI use for cognitive rehabilitation among children with cerebral palsy or learning disabilities is possible but remains challenging (Orlandi et al., 2021; Huggins et al., 2022).

### 4.5 Psychiatric disorders

EEG-based neurofeedback in ADHD, Post-traumatic Stress Disorder (PTSD), anxiety disorder, depression, obsessive-compulsive disorder (OCD), and addictions has shown promising results (Arani et al., 2010; Corominas-Roso et al., 2020; Micoulaud-Franchi et al., 2021; Chiu et al., 2022; Askovic et al., 2023; Rance et al., 2023). A meta-analysis by Chiu et al. involving 718 ADHD patients found that EEG-based neurofeedback significantly improved sustained attention but not selective attention and working memory. Similarly, fNIRS-based neurofeedback has been studied in children with ADHD (Sakurada et al., 2022). Other studies have also investigated the role of BCI in enhancing facial emotion recognition in autistic children (Smitha and Vinod, 2015; Brewe et al., 2024). Similar meta-analyses have been conducted examining the role of neurofeedback in depression and PTSD, yielding positive results (Fernández-Alvarez et al., 2022; Askovic et al., 2023).

BCIs in the form of neurofeedback lead to improvement in emotional regulation, auditory hallucinations, and reduction in the required dose of antipsychotics in patients with schizophrenia (Balconi et al., 2018; Rieger et al., 2018; Oprea et al., 2024). In a meta-analysis of 14 studies and 1,371 patients, neurofeedback significantly improved the positive (SMD = −1.05) and negative symptoms (SMD = −1.28; (Duan et al., 2025). Affective BCIs, which decode patients’ emotional responses from neural correlates and provide feedback, are also being investigated as potential therapeutic tools (Widge et al., 2014).

Despite these encouraging results, concerns remain regarding the durability of clinical benefits, as most studies had short follow-up periods. Additional concerns, such as heterogeneity in intervention protocols, small sample sizes, and the lack of control groups, currently limit broad clinical adoption of BCI in psychiatric disorders. Based on the current evidence, patients suffering from ADHD, depression, and PTSD may benefit from BCI in addition to the standard treatment.

## 5 Clinical applications of invasive and semi-invasive BCIs

Invasive BCIs have yielded remarkable results across diverse clinical conditions. Ajiboye et al., conducted a proof-of-concept study, in which a patient with chronic C4 SCI and quadriparesis received implantation of two microelectrode arrays into the left motor cortex and 36 FES electrodes over the right arm. With the help of a motorized orthosis for gravitational support, the patient was able to reach, grasp, and feed himself (Ajiboye et al., 2017). In another remarkable study, a patient with chronic C5-C6 SCI and quadriparesis received an epidural paddle lead stimulator over the root entry zone of the lumbar spinal cord, followed by a 64-channel ECoG over the bilateral motor cortices. The patient regained the ability to walk on flat surfaces and over complex terrain (stairs, steep ramps, and obstacles) with crutches (Lorach et al., 2023). Studies evaluating invasive BCIs in patients with SCI are summarized in Table 7.

**Table 7:** Summary table of studies evaluating invasive brain computer interface for motor rehabilitation of patients with spinal cord injury.

Author	Population (with sample size)	Intervention	Outcome	Number of therapy sessions	Key findings
Wang et al. (2013)	Traumatic SCI C4 AIS A 7 years since injury 1 patient	Sensor—32 contact ECoG implanted over left sensorimotor cortex Feedback –Cursor movement, control of prosthetic arm ECoG explanted after 28 days	Success rate 2D cursor control—87% 3D cursor control—80%		ECoG based BCI for cursor control
Ajiboye et al. (2017)	Traumatic SCI C4 AIS A 8 years since injury 1 patient	Sensor—2 Intracortical microelectrode arrays implanted into hand area of left sided precentral gyrus Feedback −36 percutaneous FES electrodes implanted over right upper and lower arm Arm supported by motorized arm support for gravitational assistance	Able to grasp cup of coffee and self-feed No improvement with FES turned off		Implanted FES electrodes coupled to Intracortical electrodes for arm control
Colachis et al. (2018)	Traumatic SCI C5 AIS A 7 years since injury 1 patient	Sensor—Intracortical microelectrode array implanted into hand area of left sided precentral gyrus Feedback—130 electrodes noninvasive FES over right upper and lower arm	Multi-class Classifier accuracy for 7 hand movement and rest—96.3 ± 0.7% (Paperweight) to 99.0 ± 0.5% (Hand Open) As compared to baseline, improvement in grasp and release performance even with FES off (gained ability to grasp videocassette and Can)	155–195 days	BCI use led to improvement in motor scores even with device switched off
Benabid et al. (2019)	Traumatic SCI C4-C5 1 patient	Sensor—bilateral 64 channel ECoG Feedback—Exoskeleton	Success rate for digital avatar: 2 handed, 2D tasks = 69·6% [SD 6·1] 2 handed, 3D task = 57·2% [SD 9·5] Success rate for reach and touch task mediated by exoskeleton Left hand 3D task = 68·9% [SD 1·1] Right hand 3D task = 61.5% (single experiment) No improvement in volitional motor control without BCI		ECoG based BCI for exoskeleton control
Davis et al. (2022)	Traumatic SCI C5 AIS A 6 years since injury 1 patient	Sensor—2 four contact ECoG implanted over sensorimotor cortex Feedback –mechanical glove	Median decoding accuracy—87.5 ± 4.71% Average setup time—5.6 ± 0.83 min	14 months of home use	ECoG use coupled to mechanized glove. Feasibility of extended duration home based use demonstrated.
Lorach et al. (2023)	Traumatic incomplete SCI C5-C6 10 years ago 1 patient	Sensor—bilateral 64 channel ECoG Feedback—paddle lead stimulator implanted epidurally over root entry zone of lumbar cord	Improved walking with crutches over complex terrain With stimulation off, improvement in voluntary control of hip flexor, enhanced standing and walking ability. Improvement in 6 min walk test, timed up and go, berg balance scale	40 sessions	Landmark study coupled bilateral ECoG to implanted spinal cord stimulator to support ambulation

FES, Functional Electrical Stimulation; BCI, Brain Computer Interface; SCI, spinal cord injury; ECoG, Electrocorticography; AIS, American Spinal Injury Association Impairment Scale.

Invasive BCIs have also been used to restore speech (253 electrode ECoG implanted over the left hemisphere in a patient with quadriplegia and anarthria secondary to pontine stroke. BCI was able to decode intended words and produce output through a digital avatar at a median rate of 78 words per minute and 25% error rate), touch (four patients with cervical cord injury received four intracortical microelectrode arrays each. Two arrays, each over the sensory and motor cortex. Stimulation of the electrode array implanted over the sensory cortex was paired with tactile stimulation of a bionic hand, leading to perception of touch) and vision (implantation of a microelectrode array over the right occipital cortex in a patient with complete blindness secondary to toxic optic neuropathy. Patient was able to perceive phosphenes and object boundaries; Bacher et al., 2015; Vansteensel et al., 2016; Pandarinath et al., 2017; Fernández et al., 2021; Willett et al., 2021; Chaudhary et al., 2022; Greenspon et al., 2023; Metzger et al., 2023).

Due to the inherent complexity, risks, and costs associated with these devices, most of the clinical data on a particular device is in the form of case report/ case series. Clinical trials with enrolment of multiple patients and long-term followup are available or ongoing for these devices: 96 NeuroPort Electrode (Blackrock Neurotech), Stentrode, and N1 implant (Neuralink), Responsive Neuromodulation, Adaptive Deep Brain Stimulation (Normann and Fernandez, 2016; Musk and Neuralink, 2019; Mitchell et al., 2023). The 421-channel microelectrode array, called “Connexus,” from Paradromics, and the 1,024-channel ECoG design, “Layer 7 Cortical Interface,” from Precision Neuroscience, are other commercial BCI systems in early stages of clinical testing (Precision, 2023; Connexus, 2025).

NeuroPort (initially BrainGate) is one of the earliest and most widely recognized microelectrode array in BCI clinical research. It has 96 recording micro-electrodes and was first implanted in a patient with high cervical cord injury in 2004. In the BrainGate pilot trial four patients (high cervical cord injury—two patients, brainstem stroke—one patient, ALS—one patient) underwent implantation of NeuroPort in the motor cortex for control of external devices such as cursor, robotic orthosis (Andersen et al., 2004). This trial is succeeded by the currently ongoing BrainGate2 trial (Hochberg, 2025).

The Synchron-Stentrode is an endovascular BCI with 16 electrodes implanted within a self-expanding stent. The device is positioned in the Superior Sagittal Sinus in the area adjoining the motor cortex using an endovascular approach. The connecting lead from the device crosses the internal jugular vein and is exteriorised into subcutaneous tissue in the infraclavicular area, where it connects to the implanted receiver unit. This unit then communicates to external devices. This approach reduces the surgical risk associated with a craniotomy (Oxley et al., 2016). This was the first minimally invasive device to allow ambulation and wireless control of external devices, and it received FDA “Breakthrough Device Designation” in 2020 (Han, 2021; Oxley et al., 2021). In the “SWITCH” study, Mitchell et al. reported on the safety and feasibility of using the Stentrode device. Four ALS patients with severe bilateral upper limb weakness used this device at home for over a year. At 1 year, there were no serious device-related adverse events, stent migration, or vessel occlusion. Patients were able to control a computer cursor accurately using the device (Mitchell et al., 2023). Another feasibility study called “COMMAND” is currently underway among patients with quadriplegia (Majidi et al., 2022).

The design of Neuralink’s N1 device differs significantly from the traditional microelectrode arrays. It contains 1,024 active electrodes distributed over 64 flexible leads (Neuralink, 2016). The device does not protrude beyond the level of skull and communicates wirelessly with extrinsic controllers, leading to better cosmetic outcomes and mobility. In the ongoing “PRIME” study, N1 device is being implanted in patients with quadriplegia secondary to high cervical spinal cord injury or ALS with the aim of controlling external devices So far, 21 patients have been enrolled (Neuralink, 2024a). The device has received “Breakthrough Device Designation” from FDA for restoration of vision and speech in patients with severe impairment in 2024 and 2025, respectively, (Neuralink, 2024b; Neuralink, 2025).

Invasive BCI has found its way into routine clinical practice in fields of refractory epilepsy and Parkinson’s disease (Little et al., 2013; Gummadavelli et al., 2018). In Responsive Neurostimulation, intracranial strip or depth electrodes are implanted in the suspected seizure-onset zone. Intracranial EEG is continuously monitored, and whenever an ictal rhythm is detected, electrical stimulation is automatically delivered to these electrodes, aborting the seizure. This closed-loop BCI strategy has been evaluated in clinical trial, resulting in a significant reduction in seizure frequency (−37.9% vs. − 17.3%, *p* = 0.012; Morrell and RNS System in Epilepsy Study Group, 2011). A meta-analysis of 17 studies and 541 patients found a 68% reduction in seizures and a complication rate of 18.9% (Kusyk et al., 2022).

Deep Brain Stimulation (DBS) for PD delivers electrical stimulation to the subthalamic nuclei or the globus pallidus pars interna through implanted electrodes. Typical DBS devices are not considered BCIs because they are open-loop systems, in which the clinician presets stimulation parameters in advance (Robinson et al., 2024). In Adaptive DBS (aDBS), electrical activity at the implantation site is continuously monitored. Neural activity at these sites correlates with the patient’s symptoms and is used to automatically modulate the device’s output. This closed-loop system results in better motor scores, reduced stimulation time, and lower battery consumption (Little et al., 2013). The diverse clinical applications of invasive BCIs have been comprehensively reviewed in several key papers (Levett et al., 2024; Khan et al., 2025; Lim et al., 2025).

A community survey revealed that safety and cost were the primary obstacles that would deter participants from using BCI (El-Osta et al., 2025). A recent publication analyzed the safety of intra-cortical electrodes implanted in 14 patients for the Brain Gate trial from 2004 to 2021. Over 12,203 days of BCI use, only 68 device-related adverse events were reported. Most of the adverse events were related to skin irritation (Rubin et al., 2023). Invasive BCIs carry inherent risks associated with the neurosurgical procedure. Scar formation around the intraparenchymal electrodes may increase impedance, thereby reducing the signal-to-noise ratio. However, there is accumulating evidence that the device performance remains stable over extended use (>3 years) (Pels et al., 2019). Additionally, there are risks of infection, hemorrhage, tissue damage, device failure, and lead fracture. Patients may also require additional surgery for device removal or battery replacement. Moreover, the long-term effects of implanted electrodes on brain tissue remain poorly understood. The ideal anti-thrombotic therapy for stent-based devices remains undefined, with current practice of 3 months of dual antiplatelet therapy followed by monotherapy based on clinical experience with stents (Brannigan and McClanahan, 2024). Long-term follow-up is required to evaluate the risk of thrombosis and migration in endovascular devices.

Enhanced sensors with higher signal-to-noise ratios, more active sites, lower costs, directional capabilities, and wireless transmission, along with increased computing power in compact, user-centered designs, will drive the next wave of advancements in invasive BCIs (Choi et al., 2018; Rapeaux and Constandinou, 2021). Intravascular electrode implantation at sites such as the superficial middle cerebral vein, the vein of Trolard, or Rosenthal may provide signals from critical locations to restore speech, hand function, or memory. Apart from PD and refractory epilepsy, most of the supportive literature is in the form of case series. The results from these case series, although fascinating, are limited by inherent biases, including reporting bias. Controlled studies are needed to determine real-world efficacy and feasibility across various indications. Current evidence only supports the use of adaptive DBS and responsive neurostimulation in patients with PD and epilepsy, respectively, who are not responding to medical management. The remaining invasive BCI devices are limited to clinical research.

## 6 Bridging the gap to clinics

**Figure 5:**
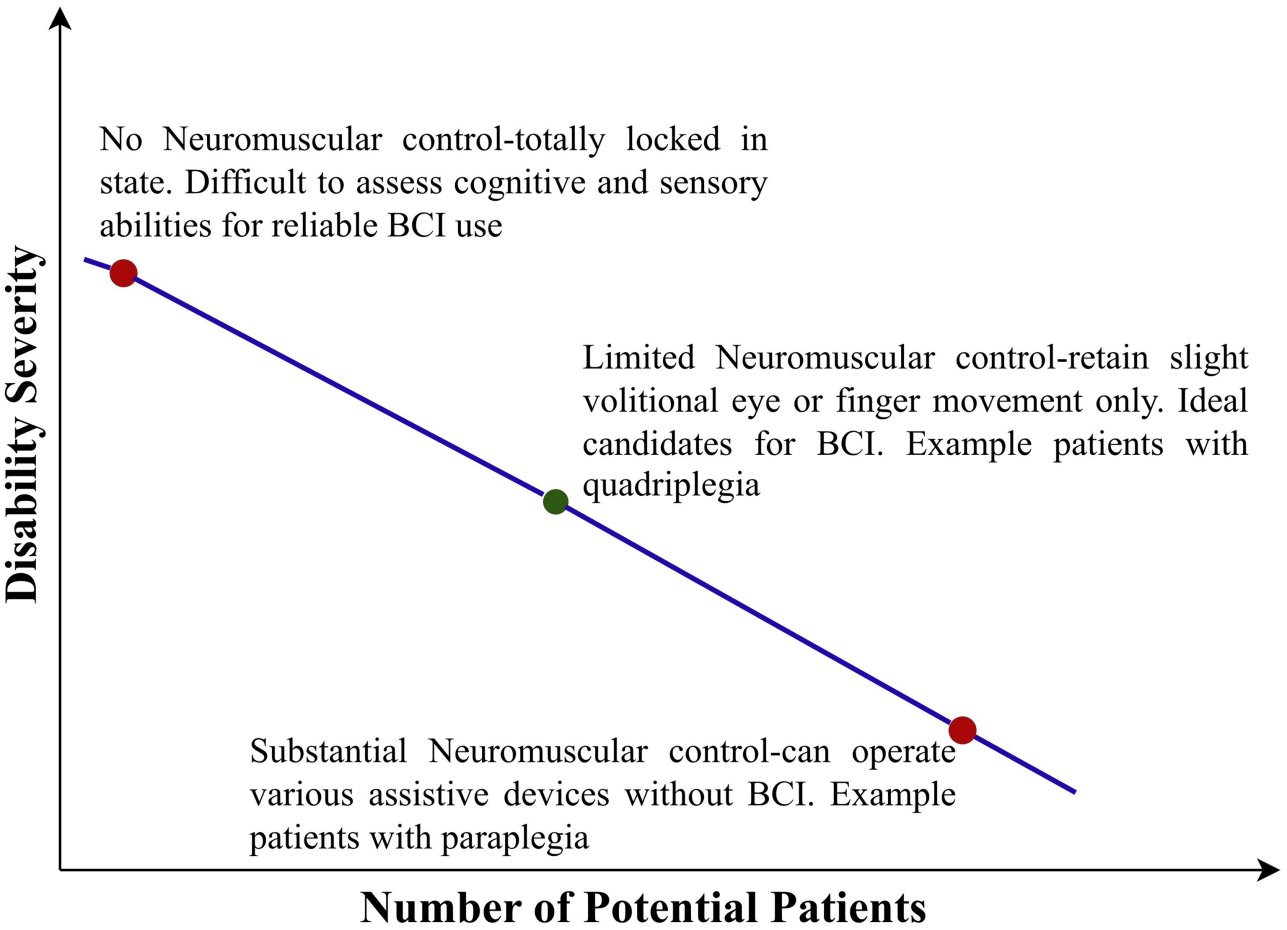
Ideal candidates for a brain computer interface at the current state of the art (BCI, Brain Computer Interface).

BCIs can alleviate various aspects of disability for an individual patient, such as motor assistance, environmental control, or communication. However, there is no consensus on when to refer patients, what level of disability qualifies them as suitable candidates, or which disease etiologies should be considered for referral. As BCI accuracy, training duration, efficacy, cost, expertise, and availability improve, the threshold for candidacy is likely to decrease. The prevailing view is that candidacy for BCI is determined by the severity of disability rather than the disease etiology (Wolpaw et al., 2006). Expert opinion on which patients make ideal candidates for BCI is summarized in Figure 5.

Evidence supporting the clinical indication should also be considered when making decisions. Table 8 summarizes the type of studies and level of evidence [based on Oxford Center for Evidence Based Medicine (CEBM)] available supporting the use of BCIs for various clinical indications.

**Table 8:** Type of studies available for various clinical indications of the brain computer interface.

Indication	Type of studies available	Oxford CEBM level of evidence	Change in clinical outcome*	Regulator approval	References
Non-invasive BCI					
Post stroke upper limb rehabilitation	Meta-analyses	Level 1	Improvement in FMA-UE SMD = 0.39–0.86	FDA approved (Ipsihand) European MDR certification (recoveriX)	(Ramos-Murguialday et al., 2013; Biasiucci et al., 2018; Cervera et al., 2018; Bai et al., 2020; Liao et al., 2023)
Post stroke lower limb rehabilitation	Meta-analysis	Level 2	Improvement in FMA-LE = 4.36–7.79	European MDR certification (recoveriX)	(Kruse et al., 2020; Zhao et al., 2022; Luo, 2024; Guo et al., 2025; Wan et al., 2025a)
Motor rehabilitation in multiple sclerosis	Case Series	Level 4	Increase in 6MWT by 37.3 meter (21.5–53.1)	European MDR certification (recoveriX)	(Carrere et al., 2021; Sebastián-Romagosa et al., 2025)
Motor rehabilitation in Parkinsons disease	Case Series	Level 4	Improvement in Timed Up and Go by 0.92 to 1.97 s	No	(Subramanian et al., 2011; Turconi et al., 2014)
Motor rehabilitation in spinal cord injury	Meta-analysis	Level 2	Improvement in LEMS by 1.4–3.5	No	(Nicolelis et al., 2022; Sun et al., 2025; Hu et al., 2026)
Motor assistive devices	Case Series	Level 4	-	No	(Aloise et al., 2011; Contreras-Vidal and Grossman, 2013; Kosmyna et al., 2016; López-Larraz et al., 2016; Meng et al., 2016; Waytowich and Krusienski, 2017)
Spellers	Case Series	Level 4	-	No	(Farwell and Donchin, 1988; Blankertz et al., 2006; Wolpaw et al., 2018; Li et al., 2021; Fodor et al., 2024)
Prolonged disorders of consciousness classification and prognostication	Case Series	Level 4	-	No	(Forgacs et al., 2014; Claassen et al., 2016; Gibson et al., 2016; Curley et al., 2018)
Dementia diagnosis and rehabilitation	Case Series	Level 4	Improvement in MoCA by 1.6–1.89 points	No	(Kober et al., 2015; Luijmes et al., 2016; Surmeli et al., 2016; Jirayucharoensak et al., 2019; Kaimara et al., 2020; Kotov et al., 2020; Galvin-McLaughlin et al., 2022; Rutkowski et al., 2024; Chen et al., 2025; Wan et al., 2025b)
Therapy for attention deficit hyperactivity disorder	Meta-analyses	Level 2	Decrease in inattention score SMD = 0.04–0.48	No	(Strehl et al., 2017; Lim et al., 2019; Van Doren et al., 2019; Neurofeedback Collaborative Group, 2021; Chiu et al., 2022; Fan et al., 2022; Lam et al., 2022; Westwood et al., 2025)
Therapy for autism	Randomized Controlled Trials	Level 3	Decrease in mean CARS-2 = 1.24	No	(Smitha and Vinod, 2015; Brewe et al., 2024; Wang et al., 2024)
Therapy for schizophrenia	Meta-analysis	Level 3	Decrease in positive symptoms (SMD = 1.05) Decrease in negative symptoms (SMD = 1.28)	No	(Balconi et al., 2018; Rieger et al., 2018; Oprea et al., 2024; Duan et al., 2025)
Invasive BCI					
Invasive BCI for motor assistance in stroke, spinal cord injury and amyotrophic lateral sclerosis patients	Case Series	Level 4	-	FDA “Breakthrough Device Designation” (NeuroPort microarray, Stentrode, ARC-BCI) FDA 510(k) (Layer 7 Cortical Interface)	(Ajiboye et al., 2017; Lorach et al., 2023)
Invasive BCI for anarthria	Case Series	Level 4	-	FDA “Breakthrough Device Designation” (Neuralink) FDA “Investigational Device Exemption” (Connexus)	(Jarosiewicz et al., 2015; Metzger et al., 2023)
Invasive BCI for refractory epilepsy (Responsive Neurostimulation)	Meta-analyses	Level 2	Mean seizure reduction rate 37.9–67%	FDA approved (NeuroPace RNS)	(Heck et al., 2014; Razavi et al., 2020; Kusyk et al., 2022; Skrehot et al., 2023; Sharma et al., 2025)
Invasive BCI for Parkinson’s Disease (Adaptive DBS)	Randomized Controlled Trials	Level 3	Decrease in most bothersome symptoms by 16.3% compared to conventional DBS	FDA approved (BrainSense)	(Bronte-Stewart et al., 2024; Oehrn et al., 2024)

*The data shown in the “Change in Clinical Outcome” column were obtained from the cited studies. Values reflect the range of effect sizes reported in high-quality studies and meta-analyses. For overall summary effect sizes, readers should consult the meta-analyses referenced in the manuscript. FDA, Food and Drug Administration, United States of America; MDR, Medical Device Regulation; Oxford CEBM, Oxford Center for Evidence-Based Medicine; CARS-2, Childhood Autism Rating Scale Second Edition; SMD, Standardized Mean Difference; FMA-UE, Fugl Meyer Assessment Upper Extremity; FMA-LE, Fugl Meyer Assessment Lower Extremity; 6MWT, 6 min walking test; sec, seconds; LEMS, Lower Extremity Motor Score; DBS, Deep Brain Stimulation. The names of regulators-approved devices in the given field are listed in brackets. Effect sizes are based on data from representative studies.

After deciding to offer BCI to a patient, the type of BCI should be determined next. When choosing, a balance must be achieved between patient needs, the quality of the neural signal required, and the risks associated with the proposed BCI device (Leuthardt et al., 2021). Patients with significant disabilities require high-performance devices with complex outputs, such as the ability to control a robotic arm. These devices require accurate neural signals for optimal performance, which are likely to come from an invasive BCI, increasing the associated risks. The use of high-risk invasive devices should only be justified for patients with substantial needs, rather than those with less severe disabilities or needs.

**Figure 6:**
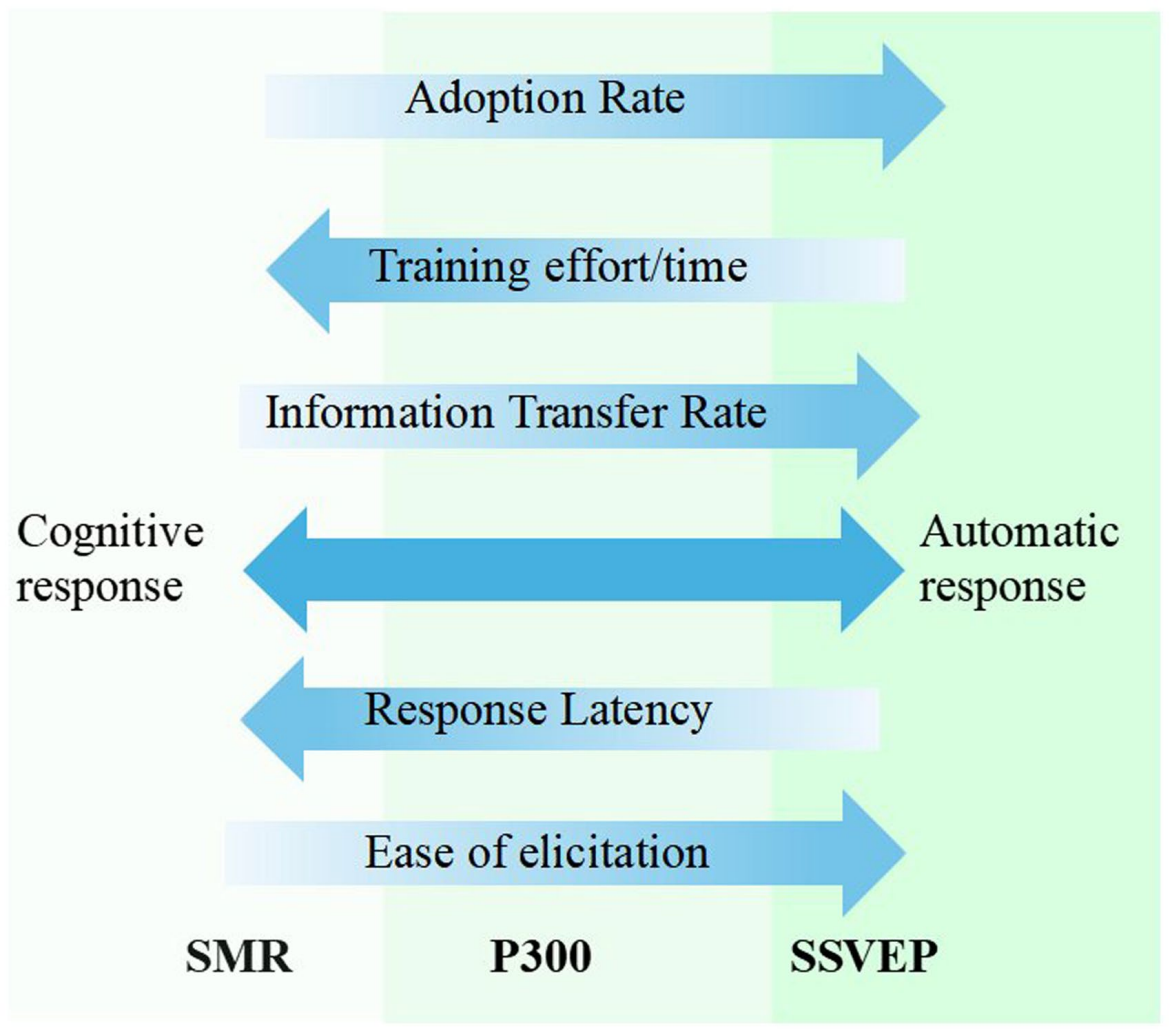
Characteristics of various paradigms in the context of spellers. SMR, sensorimotor rhythm; SSVEP, steady-state visual evoked potential.

After deciding between invasive and non-invasive BCIs, a specific BCI paradigm must be chosen. As evidenced by various speller designs, different paradigms can be used to achieve the same goal. Personalized BCI may enhance performance and improve patient experience (Ma et al., 2022). Among non-invasive BCIs, EEG and fNIRS-based BCIs are currently the only viable options outside of a laboratory setting. Notably, the availability and development of EEG-based BCIs have significantly outpaced those of fNIRS-based BCIs. Several factors influence the selection and successful deployment of BCI:

- Patient-related factors: Patient age, motivation, residual abilities (cognition, vision, hearing, ability to sit), cultural background, and social support affect BCI acceptance (Horowitz et al., 2021b; Xia and Yang, 2024). Elderly patients, limited motivation, and insufficient social support hinder BCI adoption. Medical devices/procedures (ventilator, multiple intravenous lines, nasogastric tube, external ventricular drain, hemicraniectomy) may raise safety concerns or risk of artifacts. Paradigm selection depends on the clinical scenario and the patient characteristics. Patients with poor attention or learning abilities may struggle to achieve sufficient accuracy in controlling SMR-based paradigms. Patients with low visual acuity may be better suited for SMR or P300-based paradigms. MRI-based paradigms (fMRI, MEG) require a cooperative patient and may not be suitable for children or uncooperative patients. fNIRS is less sensitive to motion artifacts and may be more reliable than EEG in studies where the patient may not be stationary and poor temporal resolution is acceptable.
- Device-related factors: Evidence supporting the proposed paradigm: Sufficient data to compare the efficacy of various paradigms is only available for restorative BCIs in patients with stroke. Paradigms that incorporate the intention of movement and FES have demonstrated higher effect sizes (Mansour et al., 2022).
  In the context of spellers, the characteristics of various paradigms are summarized in Figure 6 (Horowitz et al., 2021a; Masoodhu Banu et al., 2021). The integration of selection enhancement algorithms, such as word prediction and error correction, may improve performance. P300-based paradigms are popular, but as the number of options increases, selection time may increase. Hybrid paradigms based on SSVEP or P300 may yield more favorable outcomes.
- Logistical considerations (cost, availability, and feasibility): The number and quality of electrodes significantly impact the setup’s price. Research-grade equipment is considerably more expensive than commercial-grade equipment. Commercial-grade EEG ranges from $250 to $2,000, whereas fNIRS varies from $10,000 to $300,000 (Quaresima and Ferrari, 2019). Invasive BCIs are significantly more expensive. Robotic prostheses alone may cost over $50,000, and the surgical procedure ranges from $30,000 to $100,000 (Reilly, 2020). Additional costs for electrodes, device maintenance, and support further increase the cost of care. Because of the high initial cost, devices such as MEG, fMRI, and fNIRS are available only at a few facilities. Moreover, in low- and middle-income countries, most devices are imported from the West, thereby increasing costs. Low-cost, “do-it-yourself” options have been described in the literature, which may improve adoption.

**Figure 7:**
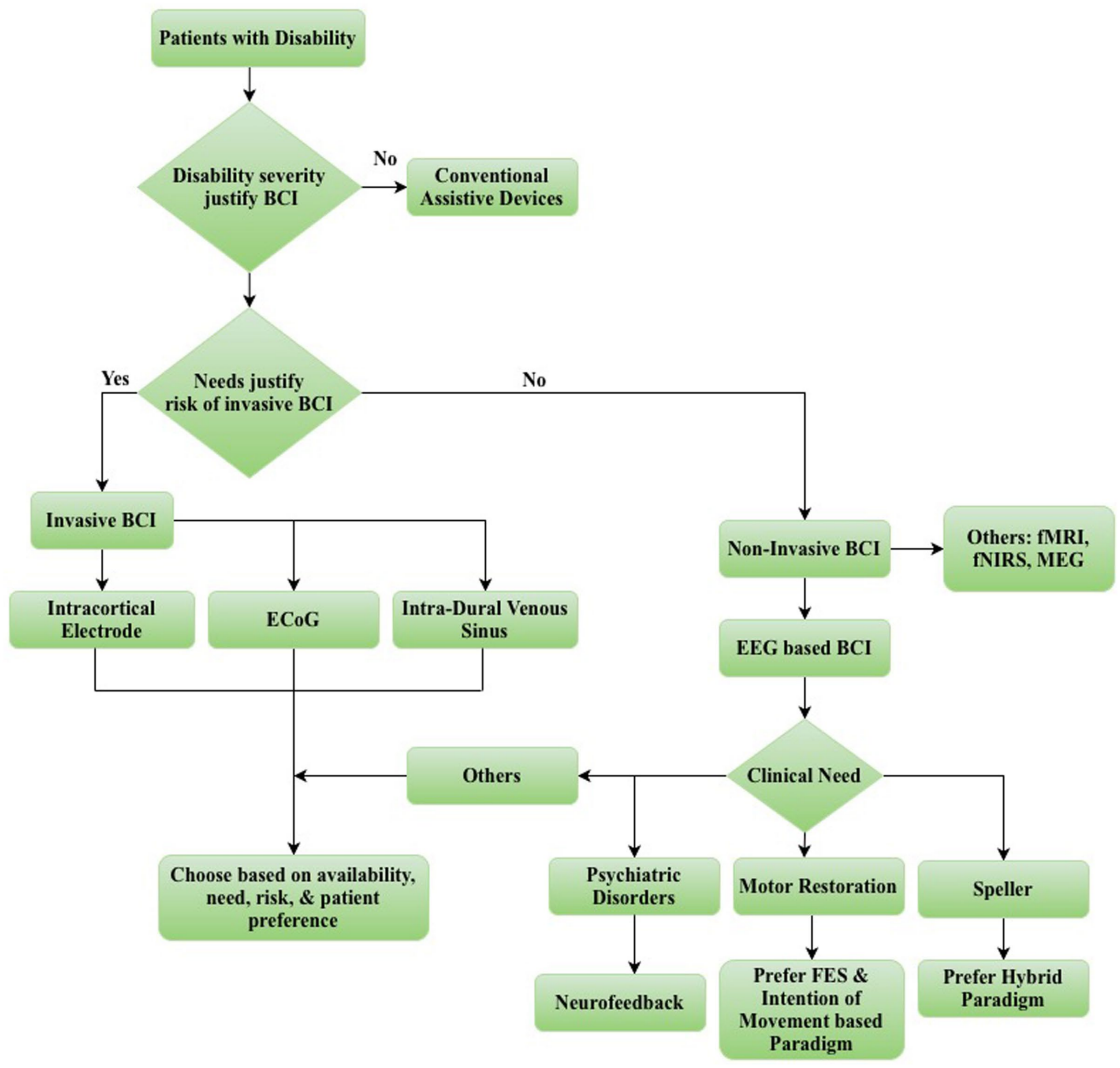
Proposed flowchart for clinical decision-making in the Brain Computer Interface (BCI, Brain Computer Interface; ECoG, Electrocorticography; EEG, Electroencephalography; FES, Functional Electrical Stimulation; MEG, Magnetoencephalography; fMRI, functional Magnetic Resonance Imaging; fNIRS, functional Near Infrared Spectroscopy).

Physicians should ensure that patients and caregivers receive adequate training and motivation to actively participate. Additionally, they should establish sufficient support systems, such as virtual troubleshooting or home visits, to facilitate effective care delivery. The proposed clinical decision-making pathway is summarized in Figure 7.

## 7 Challenges

**Figure 8:**
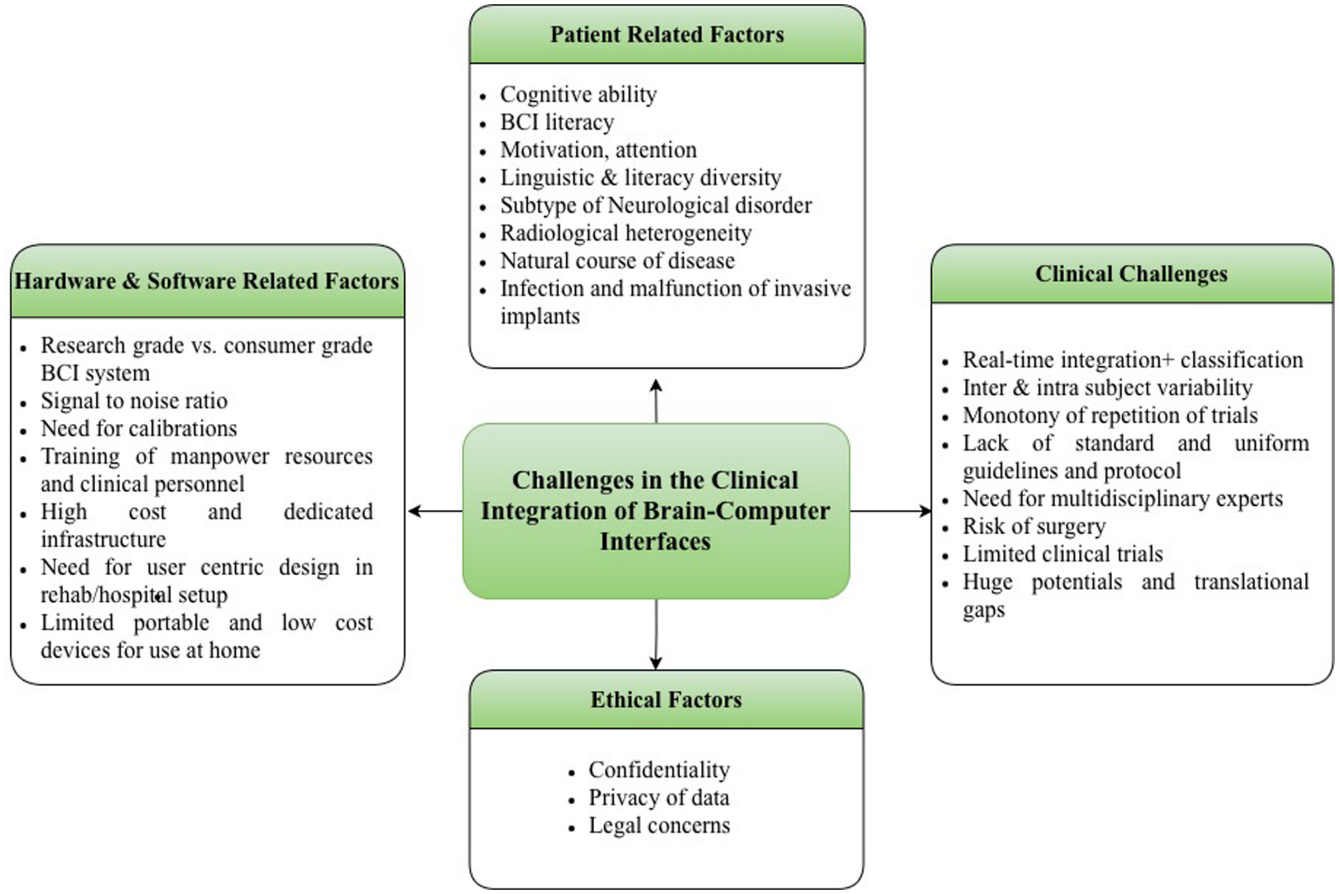
Challenges in the clinical integration of brain-computer interfaces (BCI, Brain Computer Interface; RCT, Randomized Controlled Trials).

Although the field of BCI has grown exponentially, clinical integration is still in its early stages and faces multiple challenges (Figure 8). Using BCIs requires intensive training. Despite sufficient training, up to 30% of users may struggle to interact with the interface with the necessary accuracy, a phenomenon known as BCI illiteracy (Becker et al., 2022). The system’s accuracy remains a challenge, even with state-of-the-art technology. Most published work is conducted in a highly controlled laboratory environment. Performance in the real world is expected to be lower, especially in artifact-prone EEG-based BCIs. Signal processing algorithms are increasingly adopting an artificial intelligence (AI)-based approach, which is prone to overfitting. Another concern is the inconsistency in performance between sessions or during a single session, even with a similar setup (Wolpaw, 2007). BCI use requires sustained attention, which may be challenging for some patients. The time required for “donning” (preparing the scalp, applying electrodes, and applying gel) is another major inconvenience in EEG-based BCIs.

The evidence summary tables (Tables 2–7) show that the supporting evidence for most clinical indications is limited to small-effect-size case series. This leads to a high risk of reporting bias. Existing studies are limited by small sample sizes, lack of blinding and controls, heterogeneity in BCI protocols, treatment durations, disease subtypes, and the time since disease onset. Several high-quality RCTs covering various clinical indications are necessary. Regulatory oversight is necessary to prevent centers from exploiting patients with ineffective methods while also ensuring that progress is not hindered.

The distinction between devices classified as BCIs and those that are not can sometimes be ambiguous. Some experts believe that on-device processing of brain activity is a crucial component for a device to be classified as a BCI (Robinson et al., 2024). However, this criterion is also open to interpretation. Additionally, some experts view neurofeedback as a part of BCIs (Mahrooz et al., 2024).

### 7.1 Ethical concerns

With the use of BCI, the boundaries between humans and machines are becoming blurred. This raises several ethical concerns. All BCI devices gather data on a person’s brain activity and could infringe on personal thoughts. Future BCIs might interfere with users’ autonomy by directly affecting their thoughts or actions. This raises questions about personal freedom and the danger of manipulating users’ thoughts and actions. Signals from the brain to the computer interface can also potentially be intercepted. This necessitates robust methods to ensure data security (Klein, 2020). Access to BCI may be limited by cost, region, or health care setting, worsening health inequalities (Waisberg et al., 2024).

## 8 Future directions

Motor restoration is an essential goal of BCI. Researchers need to explore the role of BCI beyond chronic stroke, including acute stroke, MS, PD, traumatic brain injury, and SCI, among others. BCIs should aim to provide holistic care to individual patients by addressing multiple aspects of their disability, such as hemiparesis, cognitive impairment, and depression, which often occur simultaneously. Most studies evaluating BCI have concentrated on “reading” from the cortex. However, “writing” BCI could improve neuroplasticity, memory, vision, and hearing, among other functions. Affordable devices for home use or smartphone-based applications, tele-neurology may help reduce healthcare disparities (González-España et al., 2025). The development of remote support systems for BCI home users will reduce the cost of care, increase BCI use, and improve performance. Such “Tele-BCI” support systems are essential for translation to clinical practice (Geronimo and Simmons, 2020). Open-source hardware designs, data, and signal processing algorithms will enable broader access to this technology.

As the technology matures and approaches clinical integration, high-quality data from randomized controlled trials becomes paramount. Consensus protocols for BCI administration and efficacy assessment should be established by international societies to facilitate the comparison of results from various trials. Additionally, tracking the response to BCI therapy through changes in brain connectivity, using diffusion tensor imaging, or repetitive transcranial magnetic stimulation, can provide valuable information. These trials should also incorporate patient-reported outcomes such as perceived usefulness, ease of use, satisfaction scores (e.g., QUEST), and safety monitoring (Demers et al., 1996). Furthermore, the burden on the user and their family can also be quantified using workload indices and donning time.

## 9 Conclusion

BCI is an interface (invasive or non-invasive) that records signals (electric, magnetic, or metabolic) from the brain and processes these signals to generate device output. The device output can “replace” speech in patients with brainstem stroke, or “restore” control over paretic muscles by coupling the BCI output with FES. BCI output can “enhance” cognition with neurofeedback training, as demonstrated among ADHD/dementia patients. BCI output can “supplement” a person by controlling a robotic arm. BCI output can also “improve” the natural brain output, as shown in rehabilitative BCIs. Applications of BCI have been explored in nearly all aspects of clinical neuroscience, such as motor impairment, communication disorders, epilepsy, cognition, PDOC, and psychiatry. Clinical integration has become feasible in post-stroke motor restoration and communication devices. Despite impressive advancements, considerable progress is needed for BCI to fulfill its potential. Future research should address shortcomings in accuracy, ITR, training time, safety, and reliability. As BCI becomes more commercially accessible, clinicians should take the lead in its further development. They can provide valuable feedback, generate evidence of its efficacy, educate patients, and promote its adoption among eligible patients.
