# Supplementary material for: Brain-computer interface: an update for the clinicians
Source: Front Hum Neurosci. 2026 Apr 22;20:1777024. doi: 10.3389/fnhum.2026.1777024 (PMC13144110; doi:10.3389/fnhum.2026.1777024)
Supplement: Supplementary file 1 [file Table_1.DOCX]

**Supplementary File**

Box 1: List of keywords used for literature review

Brain Computer Interface Communication

Brain Machine Interface Dementia

Motor rehabilitation Alzheimer

Stroke rehabilitation Speller

Parkinson disease Environment control

Duchenne Muscular Dystrophy Psychiatric disorders

Spinal-Muscular Atrophy Autism

Non-invasive BCI Neurofeedback

Sensori-motor rhythm Invasive BCI

Steady state visual evoked potential Schizophrenia

P300 Attention deficit hyperactivity disorder

Movement related cortical potential Disorders of consciousness

Electroencephalography Rehabilitative BCI

Functional near infrared spectroscopy Intracortical electrode

Magnetoencephalography Electro-corticography

Stereotactic electroencephalography Stentrode

**Included**

Full texts reviewed for eligibility

274 references included in the review

**Identification**

**Screening**

Records identified in database search:

Pubmed (n=6037

Scopus (n=7893)

PEDro (n=22)

Title and abstract screened

Records excluded as per exclusion criteria:

Preclinical studies

Animal studies

Physiology research

Non-clinical applications

Full-length articles not available in English

Duplicate records removed

Figure 1: Flowchart illustrating the study selection process.
